# Outcomes of anatomic versus reverse shoulder arthroplasty for B2 & B3 glenoids with an intact rotator cuff: An updated systematic review and proportional meta-analysis

**DOI:** 10.1177/17585732251359590

**Published:** 2025-07-17

**Authors:** Hasan Aleisawi, Colin Kruse, Nicholas Nucci, Nasser Alturki, Hassaan Abdel Khalik, George S Athwal, Moin Khan

**Affiliations:** 1 Division of Orthopaedic Surgery, Department of Surgery, 3710 McMaster University, Hamilton, ON, Canada; 2 Division of Orthopaedic Surgery, Department of Surgery, 6363 University of Ottawa, Ottawa, ON, Canada; 3 Department of Orthopedic Surgery, 37853 Prince Sultan Military Medical City, Riyadh, Kingdom of Saudi Arabia; 4 Division of Orthopaedic Surgery, Department of Surgery, 6221 Western University, London, ON, Canada

**Keywords:** reverse arthroplasty, shoulder arthroplasty, Walch classification, osteoarthritis, shoulder, total shoulder arthroplasty

## Abstract

**Background:**

It is unclear whether anatomic total shoulder arthroplasty (aTSA) or reverse total shoulder arthroplasty (rTSA) produces better outcomes in patients with Walch type B2 and B3 glenoids. The purpose of this systematic review was to examine the clinical and functional outcomes of anatomic and reverse shoulder arthroplasty in patients with B2 or B3 glenoids and preserved rotator cuff musculature.

**Methods:**

A comprehensive literature search of MEDLINE, EMBASE, and COCHRANE library was performed from the database inception through November 12, 2023.

**Results:**

There were 36 studies included in the final analysis. A total of 1349 shoulders underwent aTSA, while 478 shoulders underwent rTSA. The mean active range of motion improvements for patients undergoing aTSA and rTSA were similar, as were improvements in American Shoulder and Elbow Surgeons, Constant and Visual Analogue Scale scores. The pooled complication rate for aTSA was 7.8% (95% CI, 4.7%–11.4%) and for rTSA was 4.4% (95% CI, 2.8%–6.9%). For revision rates, aTSA had a pooled rate of 5.2% (95% CI, 3.3%–7.1%) whereas rTSA had a revision rate of 1.6% (95%CI, 1.1%–3.8%).

**Discussion:**

Based on the available data, both aTSA and rTSA effectively improved range of motion and patient-reported outcomes in patients with Walch B2 and B3 glenoids. However, aTSA demonstrated a higher complication rate (7.8% vs 4.5%) and a higher revision rate (5.2% versus 1.6%) compared to rTSA. Given the potential for bias in the included studies, these findings should be interpreted with caution.

**Level of Evidence:**

III

## Introduction

Glenohumeral osteoarthritis is a degenerative disease of the shoulder that imposes a significant burden on healthcare systems both in North America and globally, with profound financial and quality of life costs. In the United States alone, over 100,000 total shoulder arthroplasties are performed annually, and the rate has increased dramatically over the past two to three decades.^1^ In North America, the direct and indirect costs of osteoarthritis treatment is well into the billions of dollars and continues to rise with aging populations.^1^ As the disease progresses and surgical reconstruction becomes necessary, anatomic total shoulder arthroplasty (aTSA) and reverse total shoulder arthroplasty (rTSA) emerge as the primary surgical interventions.

Preoperative glenoid morphology has been shown to influence outcomes following shoulder arthroplasty, including rates of loosening, revision, and patient-reported outcomes.^2^ The Walch classification utilizes axial imaging to categorize glenoids into four main types. Of interest to this review, Walch B2 and B3-type glenoids pose challenges to the treating surgeon due to the severity of the glenoid deformity, posterior glenoid erosion, retroversion, and posterior humeral head subluxation.^3^ In primary total shoulder arthroplasty, strategies such as bone grafting (BG), eccentric reaming (EccR), and the use of posteriorly augmented glenoid (PAG) components have been developed to deal with abnormal glenoid morphology.^4–7^ While anatomic replacements in shoulders with biconcave glenoids may yield positive clinical outcomes, the procedure is associated with a higher rate of complications, particularly glenoid component loosening.^7,8^ Comparatively, rTSA appears to have a lower glenoid-sided complication rate.^9^ Recently, rTSA indications have expanded to include patients with intact rotator cuffs and end-stage osteoarthritis with Walch B2 and B3 erosions.^9–14^ Both aTSA and rTSA have proven efficacy, improving patient-reported outcome measures (PROMs), pain and range of motion (ROM), however, the optimal surgical strategy remains undetermined, particularly in the treatment of B2 and B3-type glenoid deformities.^15^ Reahl et al.^14^ performed a robust systematic review and meta-analysis on 16 studies focusing on the Walch B2 glenoid treated with aTSA or rTSA. However, many of these studies are relatively outdated, and the full spectrum of the pathology was not adequately depicted, as they did not include B3 glenoids. A systematic review was conducted by Heifner et al.^9^ on rTSA in patients with an intact rotator cuff; however, a comparison with aTSA was not included, and the focus on B2 and B3 glenoids was lacking. Thus, the purpose of this paper was to examine the ROM, PROMs, complications and revision rates of anatomic and reverse shoulder arthroplasty in patients with B2 or B3 glenoids and preserved rotator cuff musculature.

## Methods

**Figure 1.**
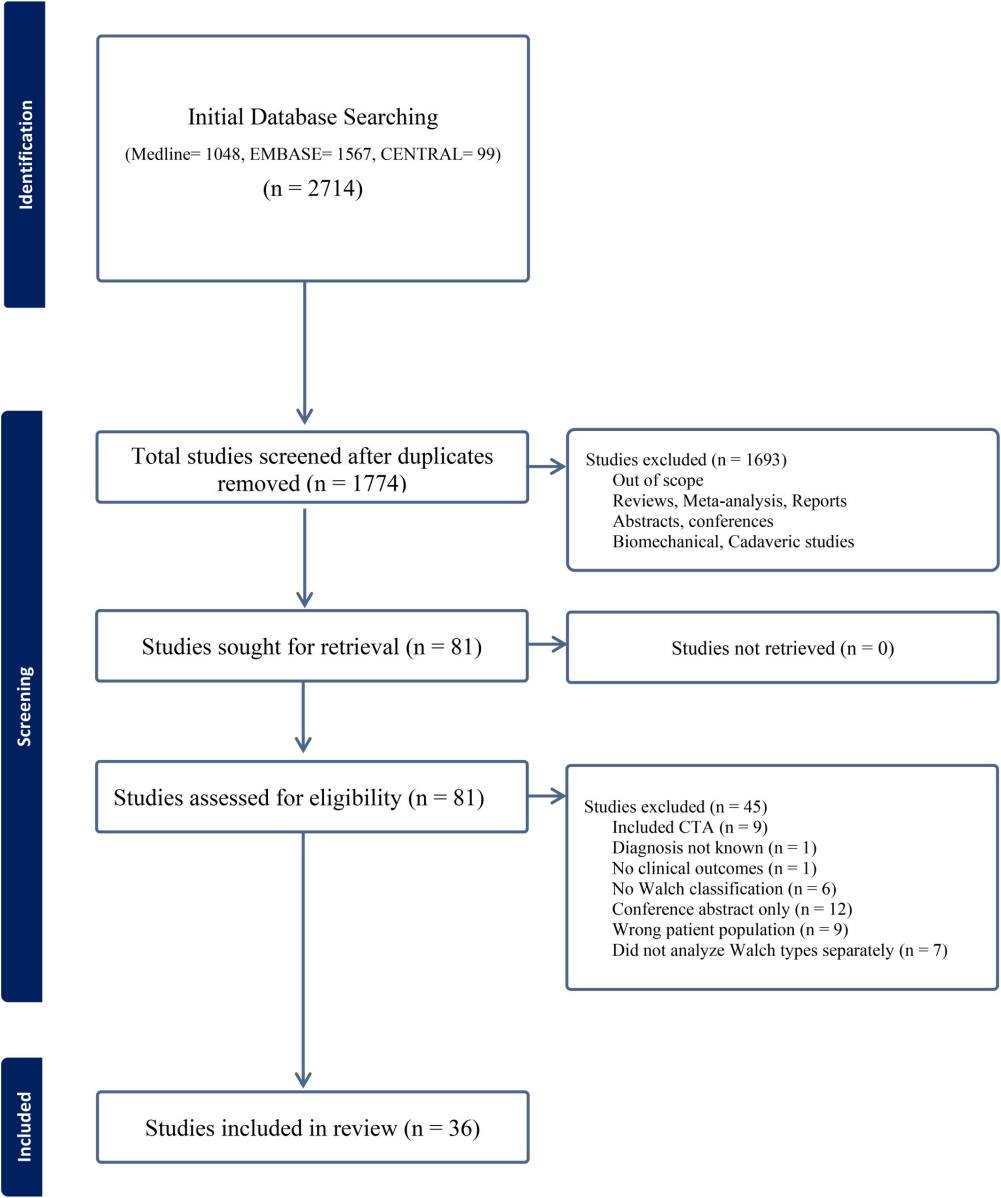
Preferred Reporting Items for Systematic Reviews and Meta-Analyses (PRISMA) diagram of the included studies.

This systematic review was conducted in concordance with the Cochrane Handbook for Systematic Reviews of Interventions and reported in accordance with the Preferred Reporting Items for Systematic Reviews and Meta-Analyses (PRISMA) guidelines (Figure 1).^16,17^

### Eligibility criteria

#### Comprehensive search strategy

Three electronic databases, MEDLINE, EMBASE, and COCHRANE library, were electronically searched from the database inception through November 12, 2023. The search strategy incorporated a combination of terms related to ‘reverse shoulder arthroplasty’, ‘anatomic shoulder arthroplasty’ and ‘B2/B3 glenoid’. A grey literature search was also conducted to ensure the inclusion of online articles published ahead of print were included.

The inclusion criteria were defined as follows: (1) studies investigating primary aTSA; (2) studies investigating primary rTSA; (3) studies focusing on B2 glenoid morphology; (4) studies focusing on B3 glenoid morphology; (5) studies reporting postoperative outcome measures, such as ROM, ASES, Constant score, complications, and revisions; (6) a minimum follow-up period of one year; and (7) studies published in English.

The exclusion criteria included: (1) Studies that analysed revision cases; (2) studies with no extractable data on B2/B3 glenoid morphologies; (3) studies with rotator cuff pathology; (4) studies on hemiarthroplasty; (5) non-English articles; (6) cadaveric studies; and (7) narrative reviews and case reports.

#### Study screening

Study screening was conducted independently by two authors (HA and NA) in accordance to the predefined eligibility criteria using Covidence (Covidence systematic review software, Veritas Health Innovation, Melbourne, Australia). Discrepancies at the title/abstract stage were advanced to full-text review to avoid any premature exclusions. Disagreements at the full-text stage were resolved through consensus. If consensus could not be reached, a third senior author was consulted (MK). Manual screening of the references of included studies was also performed to identify additional articles. Inter-reviewer agreement at each stage of the screening process was calculated using Cohen's Kappa (κ). Agreement was categorized a priori as: κ of 0.81–1.0 indicated near-perfect agreement; κ of 0.61–0.80 indicated substantial agreement; κ of 0.41–0.60 indicated moderate agreement; and κ of 0.21–0.40 indicated fair agreement.^18^ Interobserver agreement for methodological quality assessment was calculated using the intraclass correlation coefficient, with a value of ≥ 0.65 considered adequate.^19^

#### Assessment of study quality

Three researchers independently assessed the risk of bias and quality of the studies using the Methodological Index for Non-Randomized Studies (MINORS).^20^ The MINORS scale assigns a score of 0, 1, or 2 for a list of 8 questions in non-comparative studies and 12 questions in comparative studies. Any discrepancies between reviewers were resolved through discussion.

#### Data abstraction

Data were independently abstracted by three reviewers (HA, NN, & NA) using a predefined, collaborative web-based spreadsheet (Google Sheets, 2021, California, United States: Google LLC). To ensure accuracy, all reviewers audited the results, and an additional senior author conducted a review (HAK). The extracted data encompassed basic study characteristics (authors, publication year, country, study type), patient demographics (age, gender, sample size, number of shoulder injuries, Walch classification, intervention type), method of glenoid correction and implants type when available, radiological outcomes (such as glenoid implant position, humeral head alignment, osteolysis, glenoid loosening, fracture healing), ROM, PROMs including the Constant Score, American Shoulder and Elbow Surgeons (ASES) Score, Simple Shoulder Test (SST), and Shoulder Pain and Disability Index (SPADI), as well as complications (infection, glenoid loosening, cuff tear/failure, posterior dislocation, frozen shoulder, periprosthetic fracture, revision surgery). For any uncertainties regarding the data, authors of the included studies were contacted for clarification.

Studies were further sub-categorized by technique employed to correct posterior glenoid bone loss including (1) aTSA with EccR, (2) aTSA with PAG, and (3) aTSA with non-corrective reaming (NCR).

### Statistical analysis

A proportional meta-analysis was conducted to evaluate the complication and revision rates among aTSA and rTSA, identified as the most consistent outcome variable amenable to such analysis, using a method similar to that employed in the study by Reahl et al.^14^ Pooled estimate rates were calculated using a Freeman–Tukey transformation (arcsine square root transformation) within a random-effects model, and 95% confidence intervals were estimated utilizing the DerSimonian-Laird estimator.^21^ The selection of this model was dictated by the need to accommodate variations among studies in terms of patient demographics, surgical techniques, and research methodologies.^22^

Variability among the included studies was quantified through the I^2 statistic, which estimates the percentage of total variation across studies attributable to heterogeneity rather than chance.^23^ According to guidelines from the Cochrane Review Handbook, I^2 values ranging from 0 to 40% are deemed not significant, values from 30% to 60% suggest moderate heterogeneity, those from 50% to 90% indicate substantial heterogeneity, and values from 75% to 100% are considered to reflect considerable heterogeneity.^16^ The analysis of the data was performed using Medcalc software (Oostende, Belgium).

## Results

### Study and patient characteristics

The search initially identified 2714 studies. After exclusionary criteria and removal of duplicates, 36 studies were included in the final analysis. Of these, 24 studies exclusively evaluated aTSA,^4–8,24–42^ six focused solely on rTSA,^12,13,43–46^ and six assessed both aTSA and rTSA.^10,11,47–50^

A total of 1349 shoulders underwent aTSA, with 1110 (85%) being B2 glenoids and 190 (15%) B3 glenoids (Table 1). The mean age for this group was 68 ± 8 years with 453 (37%) patients being female and mean follow-up being 4.9 ± 2.7 years. For rTSA, 478 shoulders were treated, with 304 (70%) classified as B2 glenoids and 131 (30%) as B3 glenoids (Table 2). The mean age was 72 ± 5 years with 132 (41%) patients being female with mean follow-up being 4.2 ± 2.6 years.

**Table 1.** Demographic data for included aTSA studies.

Authors	Level of evidence	Patients (shoulders), n	B2 glenoids (n)	B3 glenoids (n)	Sex ratio, M:F	Mean age at surgery (range or SD), years	Mean follow-up time (range or SD), years
Alentorn-Geli et al., 2018^a^	III	15 (15)	15	0	14:1	70.5 (±7.5)	3.56 (±1.53)
Bevan et al, 2023^a^	III	18 (18)	18	0	9:8	72 (±4)	1.25 (0.50–4.67)
Chamberlain et al, 2020	IV	20 (20)	20	0	16:4	61.7 (43–81)	4.08 (2.33–6)
Chen et al, 2020	III	22 (22)	22	0	14:8	69.8 (±8.4)	6.6 (±0.90)
Chin et al, 2015	III	48 (48)	48	0	19:29	68.7 (48–85)	5 (1.92–10)
Conyer et al, 2023	IV	30 (30)	30	0	23:7	65.4 (±6.8)	9.9 (±3.7)
Cuff et al, 2023^a^	III	101 (101)	96	5	68:33	66 (48–78)	8.33 (7–12)
Egger et al, 2019	III	15 (15)	15	0	NR	NR	NR
Favorito et al, 2016	IV	19 (22)	22	0	15:5	62 (44–77)	3 (2.17–3.83)
Gallusser et al, 2014^a^	IV	17 (19)	NR	NR	NR	66 (47–79)	4.75 (2.00–7.92)
Grantham et al, 2020	IV	43 (45)	45	0	35:8	64.6 (±6.9)	4.9 (2–10.4)
Grey et al, 2020	IV	58 (58)	46	12	19:39	64.52 (±7.49)	4.16 (±1.48)
Gutman et al, 2023	IV	50 (50)	41	9	38:12	67.6 (±8)	3.5 (2–8.83)
Habermeyer et al, 2007	II	24 (24)	24	0	NR	NR	NR
Harold et al, 2023	IV	33 (34)	34	0	NR	65.5 (38–83)	8.6 (5.5–11.2)
Hinse et al, 2023	IV	30 (32)	32	0	14:16	65.1 (36.5–77.1)	9.2 (5.5–16.6)
Ho et al, 2018	IV	71 (71)	46	25	55:16	65 (±7)	NR
Hussey et al, 2015	III	78 (78)	78	0	55:23	NR	NR
Iannotti et al, 2021	II	50 (50)	29	21	37:13	63.5 (±6.1)	2.3 (±0.4)
Klika et al, 2014	IV	11 (11)	11	0	5:6	64.63 (55–75)	8.7 (2–50)
Kohan et al, 2022	III	35 (35)	0	35	31:4	69.8 (±8.6)	8.7 (±1.79)
Leschinger et al, 2017	II	27 (27)	NR	NR	NR	NR	NR
Magosch et al, 2017^a^	IV	68 (68)	68	0	39:29	64.9 (±9.7)	4.05 (±2.3)
Matsen et al, 2020	IV	135 (135)	83	52	76:59	69.58 (±8.62)	2.84 (±1.33)
Orvets et al, 2018	IV	59 (59)	59	0	36:23	64 (38–84)	4.17 (2.00–8.08)
Pastor et al, 2015	III	4 (4)	4	0	NR	NR	NR
Polisetty et al, 2023^a^	III	101 (101)	70	31	54:47	71 (±6.3)	3.92 (2–10.17)
Sheth et al, 2020	III	111 (111)	111	0	80:31	65.9 (±8.9)	3.33 (±1.25)
Stephens et al, 2017	IV	21 (21)	19	0	NR	66 (58–81)	2.92 (2–3.42)
Walch et al, 2012	IV	75 (92)	92	0	29:46	68 (50–85)	6.42 (1.17–15)
Total		1389 (1416)	85%	15%	63%:37%	66.7	4.9

aTSA, anatomic total shoulder arthroplasty; SD, standard deviation; EccR, eccentric reaming; PAG, posteriorly augmented glenoid; BG, bone grafting; rTSA, reverse total shoulder arthroplasty.

^a^ indicates study includes both rTSA and aTSA.

**Table 2.** Demographic data for included rTSA studies.

First author & year	Level of evidence	Patients (shoulders) (n)	B2 glenoids (n)	B3 glenoids (n)	Sec ratio, M:F	Mean age at surgery (range or SD), years	Mean follow-up time (range or SD), years
Alentorn-Geli et al, 2018^a^	III	16 (16)	16	0	11:5	72.5 (±5.4)	2.93 (±1.18)
Bevan et al, 2023^a^	III	17 (19)	19	0	9:8	75 (±7)	1.5 (0.5–3.83)
Collin et al, 2019	IV	27 (27)	15	12	NR	74.2 (NR)	NR
Cuff et al, 2023^a^	III	93 (93)	69	20	68:25	69 (52–80)	7.83 (6.17–11)
Gallusser et al, 2014	IV	8 (8)	NR	NR	NR	79 (73–85)	3.58 (2–5.75)
Harmsen et al, 2017	IV	26 (29)	16	10	18:9	70.1 (46–87)	2.61 (1.98–7.41)
Magosch et al, 2017 ^a^	IV	7 (7)	7	0	3:4	70.1 (±6.7)	2.65 (±1.25)
Mizuno et al, 2013	IV	27 (27)	NR	NR	5:22	74.1 (66–82)	4.5 (2–11.58)
Pettit et al, 2022	III	106 (106)	57	49	NR	NR	NR
Pharr et al, 2021	III	32 (32)	29	2	20:12	71 (61–81	2.4 (2–3.7)
Polisetty et al, 2023 ^a^	III	101 (101)	70	31	54:47	72 (±6)	2.58 (2–7.08)
Waterman et al, 2020	III	20 (20)	13	7	NR	NR	NR
Total		480 (485)	70%	30%	59%:41%	71.6	4.2

aTSA, anatomic total shoulder arthroplasty; SD, standard deviation; EccR, eccentric reaming; PAG, posteriorly augmented glenoid; BG, bone grafting; rTSA, reverse total shoulder arthroplasty.

^a^ indicates study includes both rTSA and aTSA.

### Surgical technique utilized

Regarding aTSA, twenty studies utilized pegged all-polyethylene glenoid components,^5–7,10,11,24,28–32,34,35,38–42,47,50^ while two used keeled components.^37,48^ Two studies employed either keeled or pegged designs,^4,33^ and two studies utilized metal-backed glenoid designs.^8,25^ The implant type was not reported in three studies. Concerning the method of correction, EccR was predominantly used in 15 studies, PAGs in six, and NCR in two. Five studies did not specify the correction method, and one study used either PAG or EccR. Furthermore, additional procedures were performed in three studies, including posterior capsular plication in two, and rotator interval closure in one.

Regarding rTSA, significant variation in the implant designs used across the studies prevented a uniform report on their specifics. Regarding the method of correction, EccR, with or without BG, was used in seven studies. Three studies did not report the correction method, and one study used either augments or standard baseplates.

### Range of motion

The mean active ROM improvements for patients undergoing aTSA and rTSA were 44° and 46° in flexion, 29° and 24° in external rotation, and 55° and 54° in abduction, respectively (Table 3). For patients with B3 glenoids, the mean active ROM improvements were 52° and 44° in flexion, and 27° and 26° in external rotation for aTSA and rTSA, respectively (Table 3). Within the aTSA subgroups, the mean active ROM improvements were 42° and 48° in flexion, 28° and 30° in external rotation, and 52° and 55° in abduction for aTSA + EccR and aTSA + PAG, respectively.

**Table 3.** Comparison of ROM data for included aTSA and rTSA studies.

	Total patients (shoulders)	Mean flexion at final FU, (SD)	Δ flexion at final FU, (SD)	Mean external rotation at final FU, (SD)	Δ external rotation at final FU, (SD)	Mean abduction at final FU, (SD)	Δ abduction at final FU, (SD)
All rTSA	480 (485)	144.1 (460)	45.7 (425)	43.4 (460)	24.3 (425)	129.5 (58)	54.5 (50)
rTSA – B3	131 (131)	138 (92)	44.1 (49)	45.6 (92)	25.6 (49)	NR	NR
All aTSA	1389 (1416)	148.6 (790)	44 (626)	47.4 (916)	29.1 (737)	138 (239)	55.4 (222)
aTSA – B3	190 (190)	146.4 (87)	52.5 (21)	47 (87)	27 (21)	143 (12)	55 (12)
aTSA + ER	742 (762)	147.1 (462)	41.6 (294)	47.7 (525)	27.9 (405)	135.7 (96)	51.5 (96)
aTSA + PAG	285 (288)	152.5 (235)	47.5 (219)	44 (235)	29.5 (219)	143 (58)	55 (58)
aTSA + NCR	178 (180)	NR	NR	NR	NR	NR	NR

Δ: change; FU: follow-up; SD: standard deviation; NR: not reported; EccR: eccentric reaming; PAG: posteriorly augmented glenoid; NCR: non-corrective reaming; aTSA: anatomic total shoulder arthroplasty; rTSA: reverse total shoulder arthroplasty; ROM: range of motion.

### Patient reported outcomes

Regarding patient-reported outcomes, aTSA and rTSA demonstrated average improvements in ASES scores of 44 and 50, respectively (Table 4). Average improvements in Constant scores were 43 for aTSA and 46 for rTSA. Additionally, Visual Analogue Scale (VAS) pain scores improved by −5.7 for aTSA and −5.4 for rTSA. In the B3 cohort, aTSA and rTSA showed average improvements in ASES scores of 53 and 55, respectively.

**Table 4.** Comparison of PROMs, complication, and revision data for included aTSA and rTSA studies.

	Total patients (shoulders)	Postop ASES at final FU, points (*n*)	Δ ASES at final FU, points (*n*)	Postop CS at final FU, points (*n*)	Δ CS at final FU, points (*n*)	Postop VAS at final FU, points (*n*)	Δ VAS at final FU, points (*n*)	Complications, all, *n* (%)	Revisions, all, *n* (%)
All rTSA	480 (485)	86.1 (391)	49.7 (358)	72 (69)	46.2 (34)	0.52 (265)	−5.41 (265)	19/431 (4.4)	7/431 (1.6)
rTSA – B3	131 (131)	91 (49)	55.3 (56)	66 (12)	NR	0.44 (49)	−5.27 (56)	1/49 (2)	1/49 (2)
All aTSA	1389 (1416)	84.3 (745)	43.9 (625)	75.6 (387)	42.8 (366)	1.13 (348)	−5.79 (283)	101/1315 (7.6)	70/1329 (5.2)
aTSA – B3	190 (190)	89.87 (47)	53.1 (12)	81.3 (12)	44.7 (12)	0.78 (9)	NR	8/120 (6.6)	1/120 (0.8)
aTSA + ER	742 (762)	84.7 (543)	44.6 (503)	74.4 (284)	44.5 (284)	1.21 (213)	−5.59 (213)	63/751 (8.3)	44/778 (5.6)
aTSA + PAG	285 (288)	90.9 (95)	49.2 (79)	79 (58)	36 (58)	0.74 (90)	−5.81 (40)	14/307 (4.5)	7/307 (2.2)
aTSA + NCR	178 (180)	79.6 (43)	27.1 (43)	NR	NR	NR	NR	10/180 (5.5)	10/180 (5.5)

Δ, change; PROMs, patient-reported outcome measures; ASES, American Shoulder and Elbow Surgeons; VAS, Visual Analogue Scale; CS, constant score; FU, follow-up; SD, standard deviation; NR, not reported; EccR, eccentric reaming; PAG, posteriorly augmented glenoid; NCR, non-corrective reaming; aTSA, anatomic total shoulder arthroplasty; rTSA, reverse total shoulder arthroplasty.

### Complications and revision rates

Meta-analysis of proportions demonstrated that the pooled complication rate for aTSA was 101/1302 or 7.8% (95% CI, 4.7%-11.4%) with substantial heterogeneity (*I*² = 80.1%). In comparison, the pooled complication rate following rTSA was 19/431 or 4.4% (95% CI, 2.8%–6.9%) with no observed heterogeneity (*I*² = 0%). For revision rates, aTSA had a pooled rate of 70/1329 or 5.2% (95% CI, 3.3%–7.1%) with moderate heterogeneity (*I*² = 57.7%), whereas rTSA had a revision rate of 7/431 or 1.6% (95% CI, 1.1%–3.8%) with no heterogeneity (*I*² = 0%).

Subgroup analysis of aTSA by surgical technique demonstrated a pooled complication rate for the aTSA + EccR subgroup of 7% (95% CI, 2.9%–12.2%) with substantial heterogeneity (*I*² = 84.2%). In comparison, the aTSA + PAG subgroup demonstrated a pooled complication rate of 5% (95% CI, 1.5%–9.7%) with moderate heterogeneity (*I*² = 65.4%).

Regarding revision rates, the aTSA + EccR subgroup had a pooled revision rate of 4% (95% CI, 2.3%–7.5%) with moderate heterogeneity (*I*² = 65%). The aTSA + PAG subgroup showed a pooled revision rate of 2% (95% CI, 0.9%–5.0%) with low heterogeneity (*I*² = 26.1%). Due to the small sample size and limited studies, a proportional meta-analysis was not performed for the aTSA + NCR subgroup, which had complication and revision rates of 6%. The methodological quality of the included studies, assessed using the MINORS tool (scored out of 16 or 24, depending on study type), ranged from 9 to 20. Overall, the studies demonstrated solid methodological quality, particularly in their aims and follow-up periods.

## Discussion

The purpose of this systematic review was to compare the clinical and functional outcomes following either aTSA or rTSA in patients with symptomatic primary glenohumeral osteoarthritis and a Walch B2 or B3 glenoid morphology with an intact rotator cuff. There has recently been a shift towards using rTSA in this patient population and an associated increase in the literature. It was the goal of this review to collate and analyze this large body of new evidence. A key strength of this review is the large volume of compiled data, including a total of 1349 shoulders that underwent aTSA and 478 that underwent rTSA. As well, this review was able to perform an analysis on aTSA and rTSA in patients with B3 glenoid morphology, which has been neglected in previous reviews.^9,14^ It was hypothesized that lower revision rates would be seen in the rTSA group compared to the aTSA group, especially in patients with severe glenoid deformities. Overall, the results of this review demonstrated that both aTSA and rTSA resulted in comparable improvements in ROM and patient-reported outcomes. However, aTSA showed a higher complication rate (7.8% vs 4.5%) and a 3× higher revision rate (5.2% versus 1.6%) as compared to rTSA when all patients were pooled. Due to the single-arm nature of many of the included studies, a direct statistical comparison between the separate pooled means was not performed, however, it does appear that there is a trend favouring rTSA. These results are similar to the previous review by Reahl et al.^14^ which investigated mid-term outcomes of Walch B2 glenoids between aTSA and rTSA. The authors found pooled complication rates of 9% for aTSA and 6% for rTSA and pooled revision rates of 2% for aTSA and 1% for rTSA, which were not statistically significant differences.^14^ The current review also reviewed aTSA and rTSA in Walch B3 glenoids.^2,3^ Although limited by small cohorts and reported outcomes, results showed pooled complication rates of 7% in aTSA and 2% in rTSA in B3 patients. Interestingly, the pooled revision rates were 1% in aTSA, but 2× as high (2%) in rTSA in this subgroup (Table 4). Polisetty et al.^50^ in their 202 patient cohort of B2 and B3 glenoids reported excellent outcomes and lower complications in both aTSA and rTSA. Only two glenoid baseplates in the rTSA group showed loosening compared with 29 aTSA cases that showed glenoid radiolucencies with three being grossly loose. Pettit et al.^12^ also reported excellent clinical outcomes in patients with B3 glenoids post rTSA with only one patient experiencing baseplate failure that required revision. It has been well established that the glenoid component is disproportionally responsible for complications and revisions post aTSA.^7–9,51^ Glenoid component loosening, polyethylene wear and glenohumeral decentering all contribute to failure and the need for subsequent revision surgery.^51^ The rTSA has a semi-constrained design, minimizing the risk of posterior humeral head subluxation, and providing more robust glenoid baseplate fixation leading to decreased likelihood of glenoid component loosening.^2,14^ However, complications more commonly associated with rTSA due to its implant design include stress fractures of the acromion or scapular spine, which have been reported in approximately 4–5% of all rTSA cases.^52^ This review was not able to analyze specific complications due to lack of consistent reporting, however, it appears as though rTSA results in generally lower observed pooled complication and revision rates.

Comparing ROM and PROMs between aTSA and rTSA, the results were similar. The ROM measurements at final follow-up and the mean active ROM improvements were not statistically different, although there was a trend favouring aTSA in external rotation and abduction (Table 3). It is important to note that due to limited reporting, internal rotation was not analyzed in this review. Internal rotation plays a key role in many activities of daily living, such as personal hygiene. Some studies have indicated that aTSA may provide superior functional internal rotation compared to rTSA.^53,54^ Six studies have reported the outcomes of patients with B3 glenoids undergoing aTSA.^5,6,30,31,38,50^ The mean postoperative flexion and external rotation were 146° and 47°, respectively, similar to the overall aTSA mean postoperative ROM in this systematic review. In patients with B3 glenoid morphology undergoing rTSA, the mean postoperative flexion and external rotation were 138° and 46°, respectively. Regarding patient-reported outcomes, aTSA and rTSA demonstrated similar improvements in ASES scores, Constant scores and VAS pain scores (Table 4). The results were similar between the groups in the B3 cohort. Multiple studies demonstrated good outcomes using aTSA in patients with B3 glenoids. In his series of 9 patients with B3-type glenoids, Gutman et al. reported excellent functional improvements with augmented glenoid implants, and none exhibited glenoid loosening.^31^ The postoperative SANE score was reported as 90.6%. Similarly, Matsen et al.^38^ reported excellent functional outcomes in B3-type glenoids with a SANE score of 86%, noting that the glenoid deformity was not corrected. Kohan et al.^6^ reported outcomes on B3 glenoids with standard and augmented glenoid components, finding no difference between groups concerning functional outcomes. Cuff et al.^11^ reported one of the largest series comparing rTSA to aTSA with an intact cuff and found that patients with rTSA had a lower ASES score compared to aTSA at two years, 80 (62–90) vs 85 (68–94). However, the outcomes of aTSA were not sustained at medium-term follow-up at 7 years. rTSA continued to have an excellent ASES score while patients with aTSA demonstrated a drop in functional outcomes, 80 (60–89) vs 77 (65–88). This observation may stem from the presence of glenoid loosening that could develop during the postoperative course. Similarly, Alentorn-Geli et al.^47^ reported on aTSA vs rTSA in a small cohort of patients and showed that aTSA showed a higher ASES score compared to rTSA, 91.2 (±6.7) vs 80.3 (±14.3), respectively. Yet, two patients in the aTSA group experienced glenoid loosening, and five cases showed some degree of superior migration of the humeral head indicating cuff disease progression. None of the patients in the rTSA group experienced glenoid baseplate loosening.

This review also assessed outcomes in patients receiving aTSA based on the surgical method of correction used. EccR was found to be the most common technique utilized to correct glenoid retroversion, and it demonstrated excellent improvements in ROM and PROMs. EccR is a technique with the aim of achieving a retroversion less than 10 degrees. This is intended to enhance the backside support of the glenoid component by the native glenoid bone.^2^ However, excessive EccR can lead to medialization of the joint line, reduction in available bone stock, and detensioning of the cuff muscles.^2^ The overall rates of complications and revisions were observed to be higher than in any other correction technique group, at 7% and 4%. PAG components have been developed recently to manage certain glenoid deformities. Medialization of the joint line can be avoided, and preservation of the native glenoid bone is facilitated, along with an improvement in the backside contact of the glenoid component.^2^ Overall, the outcomes associated with augmented glenoids have been promising.^14,55^ In our systematic review, patients undergoing aTSA with a PAG demonstrated the best postoperative improvement in flexion (152.5°). The overall rates of complications and revisions for aTSA + PAG were observed to be 4.7% and 2.5%, respectively, the lowest among the different surgical methods for correction in aTSA. Outcomes were also assessed for patients undergoing aTSA with NCR, in which the glenoid was not eccentrically reamed but rather off-axis reaming was performed to obtain a monoconcave glenoid face. In this technique, a standard glenoid component was inserted without attempts to normalize the glenoid version. Matsen et al.^38^ demonstrated that the SANE score improved postoperatively at 85 for B2-type and 86 for B3-type glenoids. Grantham et al.^29^ also reported significant postoperative improvements in total ASES, ASES pain score, and SANE score, 79.6 (vs. preoperative 52.5), 41.2 (vs. preoperative 28.9), and 74.7 (vs. preoperative 52.4), respectively.

This review has several limitations. It predominantly includes studies rated as level III or IV evidence, suggesting a lower overall rigour in the study designs and potential biases in the results. As well, there was considerable heterogeneity among the included studies, particularly in regard to the use of various implants and surgical techniques within both the aTSA and rTSA groups. Grouping these differing treatment approaches under the broader categories of aTSA or rTSA introduces potential bias, as specific subgroups may demonstrate distinct outcomes. While we attempted to reduce this bias by analyzing outcomes for the different surgical methods of correction within the aTSA group, the overall low quality of evidence across the included studies presents a limitation that must be acknowledged. Additionally, there is a notably small sample size in studies concerning rTSA or aTSA for B3-type glenoids, reflecting the general scarcity of relevant literature. Furthermore, a majority of the studies lacked comprehensive reporting on crucial metrics such as PROMs, ROM, or methods of correction of glenoid deformity. This omission complicates the formulation of precise treatment guidelines for varying degrees glenoid deformities and the appropriate surgical option. In addition, significant numbers of articles were not included because they failed to detail glenoid erosion patterns or did not categorize outcomes by the specific subgroup when they did. This selective inclusion potentially narrows the scope of analyzed data.

## Conclusion

This systematic review compared aTSA to rTSA for the management of patients with B2 and B3 glenoids. The results indicated that both techniques improved ROM and patient-reported outcomes. However, aTSA showed a higher complication rate (7.8% vs 4.5%) and a higher revision rate (5.2% versus 1.6%) as compared to rTSA. In patients undergoing aTSA, the use of a PAG appears to be associated with lower complication and revision rates compared to EccR and NCR. Based on the available evidence, both aTSA and rTSA are viable options in the surgical management of patients with B2 and B3 glenoids, however, aTSA may be associated with a slightly higher complication and revision rate. Further high-quality studies are needed to validate and confirm these findings.

## Supplemental Material

sj-docx-1-sel-10.1177_17585732251359590 - Supplemental material for Outcomes of anatomic versus reverse shoulder arthroplasty for B2 & B3 glenoids with an intact rotator cuff: An updated systematic review and proportional meta-analysis Supplemental material, sj-docx-1-sel-10.1177_17585732251359590 for Outcomes of anatomic versus reverse shoulder arthroplasty for B2 & B3 glenoids with an intact rotator cuff: An updated systematic review and proportional meta-analysis by Hasan Aleisawi, Colin Kruse, Nicholas Nucci, Nasser Alturki, Hassaan Abdel Khalik, George S Athwal and Moin Khan in Shoulder & Elbow

sj-docx-2-sel-10.1177_17585732251359590 - Supplemental material for Outcomes of anatomic versus reverse shoulder arthroplasty for B2 & B3 glenoids with an intact rotator cuff: An updated systematic review and proportional meta-analysis Supplemental material, sj-docx-2-sel-10.1177_17585732251359590 for Outcomes of anatomic versus reverse shoulder arthroplasty for B2 & B3 glenoids with an intact rotator cuff: An updated systematic review and proportional meta-analysis by Hasan Aleisawi, Colin Kruse, Nicholas Nucci, Nasser Alturki, Hassaan Abdel Khalik, George S Athwal and Moin Khan in Shoulder & Elbow

sj-docx-3-sel-10.1177_17585732251359590 - Supplemental material for Outcomes of anatomic versus reverse shoulder arthroplasty for B2 & B3 glenoids with an intact rotator cuff: An updated systematic review and proportional meta-analysis Supplemental material, sj-docx-3-sel-10.1177_17585732251359590 for Outcomes of anatomic versus reverse shoulder arthroplasty for B2 & B3 glenoids with an intact rotator cuff: An updated systematic review and proportional meta-analysis by Hasan Aleisawi, Colin Kruse, Nicholas Nucci, Nasser Alturki, Hassaan Abdel Khalik, George S Athwal and Moin Khan in Shoulder & Elbow

sj-docx-4-sel-10.1177_17585732251359590 - Supplemental material for Outcomes of anatomic versus reverse shoulder arthroplasty for B2 & B3 glenoids with an intact rotator cuff: An updated systematic review and proportional meta-analysis Supplemental material, sj-docx-4-sel-10.1177_17585732251359590 for Outcomes of anatomic versus reverse shoulder arthroplasty for B2 & B3 glenoids with an intact rotator cuff: An updated systematic review and proportional meta-analysis by Hasan Aleisawi, Colin Kruse, Nicholas Nucci, Nasser Alturki, Hassaan Abdel Khalik, George S Athwal and Moin Khan in Shoulder & Elbow

sj-docx-5-sel-10.1177_17585732251359590 - Supplemental material for Outcomes of anatomic versus reverse shoulder arthroplasty for B2 & B3 glenoids with an intact rotator cuff: An updated systematic review and proportional meta-analysis Supplemental material, sj-docx-5-sel-10.1177_17585732251359590 for Outcomes of anatomic versus reverse shoulder arthroplasty for B2 & B3 glenoids with an intact rotator cuff: An updated systematic review and proportional meta-analysis by Hasan Aleisawi, Colin Kruse, Nicholas Nucci, Nasser Alturki, Hassaan Abdel Khalik, George S Athwal and Moin Khan in Shoulder & Elbow

sj-docx-6-sel-10.1177_17585732251359590 - Supplemental material for Outcomes of anatomic versus reverse shoulder arthroplasty for B2 & B3 glenoids with an intact rotator cuff: An updated systematic review and proportional meta-analysis Supplemental material, sj-docx-6-sel-10.1177_17585732251359590 for Outcomes of anatomic versus reverse shoulder arthroplasty for B2 & B3 glenoids with an intact rotator cuff: An updated systematic review and proportional meta-analysis by Hasan Aleisawi, Colin Kruse, Nicholas Nucci, Nasser Alturki, Hassaan Abdel Khalik, George S Athwal and Moin Khan in Shoulder & Elbow

sj-docx-7-sel-10.1177_17585732251359590 - Supplemental material for Outcomes of anatomic versus reverse shoulder arthroplasty for B2 & B3 glenoids with an intact rotator cuff: An updated systematic review and proportional meta-analysis Supplemental material, sj-docx-7-sel-10.1177_17585732251359590 for Outcomes of anatomic versus reverse shoulder arthroplasty for B2 & B3 glenoids with an intact rotator cuff: An updated systematic review and proportional meta-analysis by Hasan Aleisawi, Colin Kruse, Nicholas Nucci, Nasser Alturki, Hassaan Abdel Khalik, George S Athwal and Moin Khan in Shoulder & Elbow

sj-docx-8-sel-10.1177_17585732251359590 - Supplemental material for Outcomes of anatomic versus reverse shoulder arthroplasty for B2 & B3 glenoids with an intact rotator cuff: An updated systematic review and proportional meta-analysis Supplemental material, sj-docx-8-sel-10.1177_17585732251359590 for Outcomes of anatomic versus reverse shoulder arthroplasty for B2 & B3 glenoids with an intact rotator cuff: An updated systematic review and proportional meta-analysis by Hasan Aleisawi, Colin Kruse, Nicholas Nucci, Nasser Alturki, Hassaan Abdel Khalik, George S Athwal and Moin Khan in Shoulder & Elbow

sj-docx-9-sel-10.1177_17585732251359590 - Supplemental material for Outcomes of anatomic versus reverse shoulder arthroplasty for B2 & B3 glenoids with an intact rotator cuff: An updated systematic review and proportional meta-analysis Supplemental material, sj-docx-9-sel-10.1177_17585732251359590 for Outcomes of anatomic versus reverse shoulder arthroplasty for B2 & B3 glenoids with an intact rotator cuff: An updated systematic review and proportional meta-analysis by Hasan Aleisawi, Colin Kruse, Nicholas Nucci, Nasser Alturki, Hassaan Abdel Khalik, George S Athwal and Moin Khan in Shoulder & Elbow

sj-docx-10-sel-10.1177_17585732251359590 - Supplemental material for Outcomes of anatomic versus reverse shoulder arthroplasty for B2 & B3 glenoids with an intact rotator cuff: An updated systematic review and proportional meta-analysis Supplemental material, sj-docx-10-sel-10.1177_17585732251359590 for Outcomes of anatomic versus reverse shoulder arthroplasty for B2 & B3 glenoids with an intact rotator cuff: An updated systematic review and proportional meta-analysis by Hasan Aleisawi, Colin Kruse, Nicholas Nucci, Nasser Alturki, Hassaan Abdel Khalik, George S Athwal and Moin Khan in Shoulder & Elbow

sj-docx-11-sel-10.1177_17585732251359590 - Supplemental material for Outcomes of anatomic versus reverse shoulder arthroplasty for B2 & B3 glenoids with an intact rotator cuff: An updated systematic review and proportional meta-analysis Supplemental material, sj-docx-11-sel-10.1177_17585732251359590 for Outcomes of anatomic versus reverse shoulder arthroplasty for B2 & B3 glenoids with an intact rotator cuff: An updated systematic review and proportional meta-analysis by Hasan Aleisawi, Colin Kruse, Nicholas Nucci, Nasser Alturki, Hassaan Abdel Khalik, George S Athwal and Moin Khan in Shoulder & Elbow

sj-docx-12-sel-10.1177_17585732251359590 - Supplemental material for Outcomes of anatomic versus reverse shoulder arthroplasty for B2 & B3 glenoids with an intact rotator cuff: An updated systematic review and proportional meta-analysis Supplemental material, sj-docx-12-sel-10.1177_17585732251359590 for Outcomes of anatomic versus reverse shoulder arthroplasty for B2 & B3 glenoids with an intact rotator cuff: An updated systematic review and proportional meta-analysis by Hasan Aleisawi, Colin Kruse, Nicholas Nucci, Nasser Alturki, Hassaan Abdel Khalik, George S Athwal and Moin Khan in Shoulder & Elbow

sj-docx-13-sel-10.1177_17585732251359590 - Supplemental material for Outcomes of anatomic versus reverse shoulder arthroplasty for B2 & B3 glenoids with an intact rotator cuff: An updated systematic review and proportional meta-analysis Supplemental material, sj-docx-13-sel-10.1177_17585732251359590 for Outcomes of anatomic versus reverse shoulder arthroplasty for B2 & B3 glenoids with an intact rotator cuff: An updated systematic review and proportional meta-analysis by Hasan Aleisawi, Colin Kruse, Nicholas Nucci, Nasser Alturki, Hassaan Abdel Khalik, George S Athwal and Moin Khan in Shoulder & Elbow
