# Supplementary material for: Outcomes of anatomic versus reverse shoulder arthroplasty for B2 & B3 glenoids with an intact rotator cuff: An updated systematic review and proportional meta-analysis
Source: Shoulder Elbow. 2025 Jul 17;18(3):425–36. doi: 10.1177/17585732251359590 (PMC12274211; doi:10.1177/17585732251359590)
Supplement: sj-docx-1-sel-10.1177_17585732251359590 - Supplemental material for Outcomes of anatomic versus reverse shoulder arthroplasty for B2 & B3 glenoids with an intact rotator cuff: An updated systematic review and proportional meta-analysis [file sj-docx-1-sel-10.1177_17585732251359590.docx]

**Appendix Figure 1:** Forest plot of pooled complication rates of aTSA.

# Meta-analysis: proportion

| Variable for studies | Study |
| --- | --- |
| Variable for total number of cases | Total |
| Variable for number of positive cases | Complications |

| Study | Sample size | Proportion (%) | 95% CI | Weight (%) | |
| --- | --- | --- | --- | --- | --- |
|  |  |  |  | Fixed | Random |
| Alentorn-Geli et al, 2018 | 15 | 26.667 | 7.787 to 55.100 | 1.20 | 2.89 |
| Bevan et al, 2023 | 18 | 0.000 | 0.000 to 18.530 | 1.43 | 3.10 |
| Chamberlain et al, 2020 | 20 | 0.000 | 0.000 to 16.843 | 1.58 | 3.22 |
| Chen et al, 2020 | 22 | 0.000 | 0.000 to 15.437 | 1.73 | 3.33 |
| Chin et al, 2015 | 48 | 2.083 | 0.0527 to 11.070 | 3.69 | 4.10 |
| Conyer et al, 2023 | 30 | 20.000 | 7.714 to 38.567 | 2.33 | 3.66 |
| Cuff et al, 2023 | 101 | 0.000 | 0.000 to 3.586 | 7.68 | 4.58 |
| Favorito et al, 2016 | 22 | 9.091 | 1.121 to 29.161 | 1.73 | 3.33 |
| Gallusser et al, 2014 | 19 | 21.053 | 6.052 to 45.565 | 1.51 | 3.17 |
| Grantham et al, 2020 | 45 | 13.333 | 5.054 to 26.792 | 3.46 | 4.04 |
| Grey et al, 2020 | 58 | 3.448 | 0.420 to 11.908 | 4.44 | 4.24 |
| Gutman et al, 2023 | 50 | 2.000 | 0.0506 to 10.647 | 3.84 | 4.13 |
| Habermeyer et al, 2007 | 24 | 4.167 | 0.105 to 21.120 | 1.88 | 3.43 |
| Harold et al, 2023 | 34 | 11.765 | 3.300 to 27.450 | 2.64 | 3.79 |
| Hinse et al, 2023 | 32 | 31.250 | 16.118 to 50.008 | 2.48 | 3.73 |
| Ho et al, 2018 | 71 | 0.000 | 0.000 to 5.063 | 5.42 | 4.38 |
| Hussey et al, 2015 | 78 | 11.538 | 5.414 to 20.777 | 5.95 | 4.44 |
| Iannotti et al, 2021 | 50 | 6.000 | 1.255 to 16.548 | 3.84 | 4.13 |
| Klika et al, 2014 | 11 | 45.455 | 16.749 to 76.621 | 0.90 | 2.52 |
| Kohan et al, 2022 | 35 | 17.143 | 6.562 to 33.650 | 2.71 | 3.82 |
| Matsen et al, 2020 | 135 | 2.963 | 0.813 to 7.413 | 10.24 | 4.71 |
| Orvets et al, 2018 | 59 | 0.000 | 0.000 to 6.061 | 4.52 | 4.25 |
| Polisetty et al, 2023 | 101 | 5.941 | 2.211 to 12.483 | 7.68 | 4.58 |
| Sheth et al, 2020 | 111 | 7.207 | 3.163 to 13.708 | 8.43 | 4.62 |
| Stephens et al, 2017 | 21 | 0.000 | 0.000 to 16.110 | 1.66 | 3.28 |
| Walch et al, 2012 | 92 | 20.652 | 12.916 to 30.357 | 7.00 | 4.53 |
| Total (fixed effects) | 1302 | 6.485 | 5.221 to 7.946 | 100.00 | 100.00 |
| Total (random effects) | 1302 | 7.741 | 4.721 to 11.429 | 100.00 | 100.00 |

## Test for heterogeneity

| Q | 125.6783 |
| --- | --- |
| DF | 25 |
| Significance level | P < 0.0001 |
| I^2^ (inconsistency) | 80.11% |
| 95% CI for I^2^ | 71.56 to 86.09 |

## Publication bias

| Egger's test | |
| --- | --- |
| Intercept | 2.1236 |
| 95% CI | -0.6749 to 4.9222 |
| Significance level | P = 0.1304 |
| Begg's test | |
| Kendall's Tau | 0.2102 |
| Significance level | P = 0.1321 |
