# Supplementary material for: Outcomes of anatomic versus reverse shoulder arthroplasty for B2 & B3 glenoids with an intact rotator cuff: An updated systematic review and proportional meta-analysis
Source: Shoulder Elbow. 2025 Jul 17;18(3):425–36. doi: 10.1177/17585732251359590 (PMC12274211; doi:10.1177/17585732251359590)
Supplement: sj-docx-2-sel-10.1177_17585732251359590 - Supplemental material for Outcomes of anatomic versus reverse shoulder arthroplasty for B2 & B3 glenoids with an intact rotator cuff: An updated systematic review and proportional meta-analysis [file sj-docx-2-sel-10.1177_17585732251359590.docx]

**Appendix Figure 2:** Forest plot of pooled complication rates of rTSA.

# Meta-analysis: proportion

| Variable for studies | RTSA |
| --- | --- |
| Variable for total number of cases | Total |
| Variable for number of positive cases | Complications |

| Study | Sample size | Proportion (%) | 95% CI | Weight (%) | |
| --- | --- | --- | --- | --- | --- |
|  |  |  |  | Fixed | Random |
| Alentorn-Geli 2018 | 16 | 0.000 | 0.000 to 20.591 | 3.86 | 3.86 |
| Bevan 2023 | 19 | 0.000 | 0.000 to 17.647 | 4.55 | 4.55 |
| Cuff 2023 | 93 | 3.226 | 0.670 to 9.139 | 21.36 | 21.36 |
| Gallusser 2014 | 8 | 0.000 | 0.000 to 36.942 | 2.05 | 2.05 |
| Harmsen 2017 | 29 | 6.897 | 0.846 to 22.766 | 6.82 | 6.82 |
| Mizuno 2013 | 27 | 14.815 | 4.189 to 33.731 | 6.36 | 6.36 |
| Pettit 2022 | 106 | 2.830 | 0.587 to 8.049 | 24.32 | 24.32 |
| Pharr 2021 | 32 | 6.250 | 0.766 to 20.807 | 7.50 | 7.50 |
| Polisetty 2023 | 101 | 3.960 | 1.089 to 9.831 | 23.18 | 23.18 |
| Total (fixed effects) | 431 | 4.540 | 2.794 to 6.926 | 100.00 | 100.00 |
| Total (random effects) | 431 | 4.540 | 2.796 to 6.680 | 100.00 | 100.00 |

## Test for heterogeneity

| Q | 7.7118 |
| --- | --- |
| DF | 8 |
| Significance level | P = 0.4621 |
| I^2^ (inconsistency) | 0.00% |
| 95% CI for I^2^ | 0.00 to 63.74 |

## Publication bias

| Egger's test | |
| --- | --- |
| Intercept | 0.4211 |
| 95% CI | -1.7322 to 2.5744 |
| Significance level | P = 0.6578 |
| Begg's test | |
| Kendall's Tau | 0.1111 |
| Significance level | P = 0.6767 |
