# Supplementary material for: Outcomes of anatomic versus reverse shoulder arthroplasty for B2 & B3 glenoids with an intact rotator cuff: An updated systematic review and proportional meta-analysis
Source: Shoulder Elbow. 2025 Jul 17;18(3):425–36. doi: 10.1177/17585732251359590 (PMC12274211; doi:10.1177/17585732251359590)
Supplement: sj-docx-3-sel-10.1177_17585732251359590 - Supplemental material for Outcomes of anatomic versus reverse shoulder arthroplasty for B2 & B3 glenoids with an intact rotator cuff: An updated systematic review and proportional meta-analysis [file sj-docx-3-sel-10.1177_17585732251359590.docx]

**Appendix Figure 3:** Forest plot of pooled revision rates of aTSA.

# Meta-analysis: proportion

| Variable for studies | Study |
| --- | --- |
| Variable for total number of cases | Total |
| Variable for number of positive cases | Revisions |

| Study | Sample size | Proportion (%) | 95% CI | Weight (%) | |
| --- | --- | --- | --- | --- | --- |
|  |  |  |  | Fixed | Random |
| Alentorn-Geli et al, 2018 | 15 | 0.000 | 0.000 to 21.802 | 1.18 | 2.16 |
| Bevan et al, 2023 | 18 | 0.000 | 0.000 to 18.530 | 1.40 | 2.43 |
| Chamberlain et al, 2020 | 20 | 0.000 | 0.000 to 16.843 | 1.55 | 2.59 |
| Chen et al, 2020 | 22 | 0.000 | 0.000 to 15.437 | 1.70 | 2.74 |
| Chin et al, 2015 | 48 | 2.083 | 0.0527 to 11.070 | 3.61 | 4.05 |
| Conyer et al, 2023 | 30 | 10.000 | 2.112 to 26.529 | 2.29 | 3.25 |
| Cuff et al, 2023 | 101 | 9.901 | 4.851 to 17.455 | 7.52 | 5.20 |
| Favorito et al, 2016 | 22 | 9.091 | 1.121 to 29.161 | 1.70 | 2.74 |
| Gallusser et al, 2014 | 19 | 10.526 | 1.301 to 33.138 | 1.47 | 2.51 |
| Grantham et al, 2020 | 45 | 13.333 | 5.054 to 26.792 | 3.39 | 3.94 |
| Grey et al, 2020 | 58 | 3.448 | 0.420 to 11.908 | 4.35 | 4.37 |
| Gutman et al, 2023 | 50 | 2.000 | 0.0506 to 10.647 | 3.76 | 4.12 |
| Habermeyer et al, 2007 | 24 | 8.333 | 1.026 to 26.997 | 1.84 | 2.88 |
| Harold et al, 2023 | 34 | 8.824 | 1.858 to 23.678 | 2.58 | 3.46 |
| Hinse et al, 2023 | 32 | 12.500 | 3.513 to 28.995 | 2.43 | 3.36 |
| Ho et al, 2018 | 71 | 0.000 | 0.000 to 5.063 | 5.31 | 4.69 |
| Hussey et al, 2015 | 78 | 3.846 | 0.800 to 10.831 | 5.83 | 4.83 |
| Iannotti et al, 2021 | 50 | 4.000 | 0.488 to 13.714 | 3.76 | 4.12 |
| Klika et al, 2014 | 11 | 18.182 | 2.283 to 51.776 | 0.88 | 1.76 |
| Kohan et al, 2022 | 35 | 0.000 | 0.000 to 10.003 | 2.65 | 3.51 |
| Leschinger et al, 2017 | 27 | 0.000 | 0.000 to 12.770 | 2.06 | 3.07 |
| Matsen et al, 2020 | 135 | 2.963 | 0.813 to 7.413 | 10.03 | 5.56 |
| Orvets et al, 2018 | 59 | 1.695 | 0.0429 to 9.086 | 4.42 | 4.39 |
| Polisetty et al, 2023 | 101 | 0.990 | 0.0251 to 5.393 | 7.52 | 5.20 |
| Sheth et al, 2020 | 111 | 5.405 | 2.009 to 11.394 | 8.26 | 5.32 |
| Stephens et al, 2017 | 21 | 0.000 | 0.000 to 16.110 | 1.62 | 2.66 |
| Walch et al, 2012 | 92 | 16.304 | 9.423 to 25.462 | 6.86 | 5.07 |
| Total (fixed effects) | 1329 | 4.983 | 3.887 to 6.280 | 100.00 | 100.00 |
| Total (random effects) | 1329 | 4.999 | 3.284 to 7.050 | 100.00 | 100.00 |

## Test for heterogeneity

| Q | 61.5131 |
| --- | --- |
| DF | 26 |
| Significance level | P = 0.0001 |
| I^2^ (inconsistency) | 57.73% |
| 95% CI for I^2^ | 35.20 to 72.43 |

## Publication bias

| Egger's test | |
| --- | --- |
| Intercept | 0.09514 |
| 95% CI | -1.8813 to 2.0716 |
| Significance level | P = 0.9218 |
| Begg's test | |
| Kendall's Tau | 0.04578 |
| Significance level | P = 0.7376 |
