# Supplementary material for: Outcomes of anatomic versus reverse shoulder arthroplasty for B2 & B3 glenoids with an intact rotator cuff: An updated systematic review and proportional meta-analysis
Source: Shoulder Elbow. 2025 Jul 17;18(3):425–36. doi: 10.1177/17585732251359590 (PMC12274211; doi:10.1177/17585732251359590)
Supplement: sj-docx-5-sel-10.1177_17585732251359590 - Supplemental material for Outcomes of anatomic versus reverse shoulder arthroplasty for B2 & B3 glenoids with an intact rotator cuff: An updated systematic review and proportional meta-analysis [file sj-docx-5-sel-10.1177_17585732251359590.docx]

**Appendix Figure 5:** Forest plot of pooled complication rates of aTSA ER

# Meta-analysis: proportion

| Variable for studies | Studies |
| --- | --- |
| Variable for total number of cases | Patients |
| Variable for number of positive cases | Complications |

| Study | Sample size | Proportion (%) | 95% CI | Weight (%) | |
| --- | --- | --- | --- | --- | --- |
|  |  |  |  | Fixed | Random |
| Bevan et al, 2023 | 18 | 0.000 | 0.000 to 18.530 | 2.49 | 6.12 |
| Chamberlain et al, 2020 | 20 | 0.000 | 0.000 to 16.843 | 2.75 | 6.34 |
| Chen et al, 2020 | 22 | 0.000 | 0.000 to 15.437 | 3.01 | 6.53 |
| Chin et al, 2015 | 48 | 2.083 | 0.0527 to 11.070 | 6.41 | 7.86 |
| Cuff et al, 2023 | 101 | 0.000 | 0.000 to 3.586 | 13.35 | 8.67 |
| Harold et al, 2023 | 34 | 11.765 | 3.300 to 27.450 | 4.58 | 7.33 |
| Hinse et al, 2023 | 32 | 31.250 | 16.118 to 50.008 | 4.32 | 7.23 |
| Hussey et al, 2015 | 78 | 11.538 | 5.414 to 20.777 | 10.34 | 8.43 |
| Kohan et al, 2022 | 35 | 17.143 | 6.562 to 33.650 | 4.71 | 7.38 |
| Orvets et al, 2018 | 59 | 0.000 | 0.000 to 6.061 | 7.85 | 8.12 |
| Polisetty et al, 2023 | 101 | 5.941 | 2.211 to 12.483 | 13.35 | 8.67 |
| Sheth et al, 2020 | 111 | 7.207 | 3.163 to 13.708 | 14.66 | 8.74 |
| Walch et al, 2012 | 92 | 20.652 | 12.916 to 30.357 | 12.17 | 8.59 |
| Total (fixed effects) | 751 | 6.843 | 5.156 to 8.870 | 100.00 | 100.00 |
| Total (random effects) | 751 | 6.799 | 2.904 to 12.169 | 100.00 | 100.00 |

## Test for heterogeneity

| Q | 75.9191 |
| --- | --- |
| DF | 12 |
| Significance level | P < 0.0001 |
| I^2^ (inconsistency) | 84.19% |
| 95% CI for I^2^ | 74.45 to 90.22 |

## Publication bias

| Egger's test | |
| --- | --- |
| Intercept | -0.06356 |
| 95% CI | -5.6453 to 5.5182 |
| Significance level | P = 0.9805 |
| Begg's test | |
| Kendall's Tau | -0.01290 |
| Significance level | P = 0.9510 |

.

Figure 6.2 Forest plot of pooled revision rates of aTSA ER

# Meta-analysis: proportion

| Variable for studies | Studies |
| --- | --- |
| Variable for total number of cases | Patients |
| Variable for number of positive cases | Revisions |

| Study | Sample size | Proportion (%) | 95% CI | Weight (%) | |
| --- | --- | --- | --- | --- | --- |
|  |  |  |  | Fixed | Random |
| Bevan et al, 2023 | 18 | 0.000 | 0.000 to 18.530 | 2.40 | 4.62 |
| Chamberlain et al, 2020 | 20 | 0.000 | 0.000 to 16.843 | 2.65 | 4.90 |
| Chen et al, 2020 | 22 | 0.000 | 0.000 to 15.437 | 2.90 | 5.16 |
| Chin et al, 2015 | 48 | 2.083 | 0.0527 to 11.070 | 6.19 | 7.36 |
| Cuff et al, 2023 | 101 | 9.901 | 4.851 to 17.455 | 12.88 | 9.16 |
| Harold et al, 2023 | 34 | 8.824 | 1.858 to 23.678 | 4.42 | 6.40 |
| Hinse et al, 2023 | 32 | 12.500 | 3.513 to 28.995 | 4.17 | 6.22 |
| Hussey et al, 2015 | 78 | 3.846 | 0.800 to 10.831 | 9.97 | 8.59 |
| Kohan et al, 2022 | 35 | 0.000 | 0.000 to 10.003 | 4.55 | 6.48 |
| Leschinger et al, 2017 | 27 | 0.000 | 0.000 to 12.770 | 3.54 | 5.74 |
| Orvets et al, 2018 | 59 | 1.695 | 0.0429 to 9.086 | 7.58 | 7.91 |
| Polisetty et al, 2023 | 101 | 0.990 | 0.0251 to 5.393 | 12.88 | 9.16 |
| Sheth et al, 2020 | 111 | 5.405 | 2.009 to 11.394 | 14.14 | 9.34 |
| Walch et al, 2012 | 92 | 16.304 | 9.423 to 25.462 | 11.74 | 8.96 |
| Total (fixed effects) | 778 | 5.231 | 3.787 to 7.020 | 100.00 | 100.00 |
| Total (random effects) | 778 | 4.580 | 2.347 to 7.507 | 100.00 | 100.00 |

## Test for heterogeneity

| Q | 37.1570 |
| --- | --- |
| DF | 13 |
| Significance level | P = 0.0004 |
| I^2^ (inconsistency) | 65.01% |
| 95% CI for I^2^ | 38.23 to 80.19 |

## Publication bias

| Egger's test | |
| --- | --- |
| Intercept | -2.0615 |
| 95% CI | -5.3381 to 1.2150 |
| Significance level | P = 0.1955 |
| Begg's test | |
| Kendall's Tau | -0.1547 |
| Significance level | P = 0.4409 |

|  |  |
| --- | --- |
