# Supplementary material for: Outcomes of anatomic versus reverse shoulder arthroplasty for B2 & B3 glenoids with an intact rotator cuff: An updated systematic review and proportional meta-analysis
Source: Shoulder Elbow. 2025 Jul 17;18(3):425–36. doi: 10.1177/17585732251359590 (PMC12274211; doi:10.1177/17585732251359590)
Supplement: sj-docx-6-sel-10.1177_17585732251359590 - Supplemental material for Outcomes of anatomic versus reverse shoulder arthroplasty for B2 & B3 glenoids with an intact rotator cuff: An updated systematic review and proportional meta-analysis [file sj-docx-6-sel-10.1177_17585732251359590.docx]

**Appendix Figure 6:** Forest plot of pooled complication rates of aTSA PAG

Meta-analysis: proportion

| Variable for studies | studies |
| --- | --- |
| Variable for total number of cases | patients |
| Variable for number of positive cases | Complications |

| Study | Sample size | Proportion (%) | 95% CI | Weight (%) | |
| --- | --- | --- | --- | --- | --- |
|  |  |  |  | Fixed | Random |
| Favorito et al, 2016 | 22 | 9.091 | 1.121 to 29.161 | 7.32 | 11.21 |
| Grey et al, 2020 | 58 | 3.448 | 0.420 to 11.908 | 18.79 | 16.16 |
| Gutman et al, 2023 | 50 | 2.000 | 0.0506 to 10.647 | 16.24 | 15.47 |
| Ho et al, 2018 | 71 | 0.000 | 0.000 to 5.063 | 22.93 | 17.02 |
| Iannotti et al, 2021 | 50 | 6.000 | 1.255 to 16.548 | 16.24 | 15.47 |
| Kohan et al, 2022 | 35 | 17.143 | 6.562 to 33.650 | 11.46 | 13.69 |
| Stephens et al, 2017 | 21 | 0.000 | 0.000 to 16.110 | 7.01 | 10.96 |
| Total (fixed effects) | 307 | 4.150 | 2.230 to 6.988 | 100.00 | 100.00 |
| Total (random effects) | 307 | 4.760 | 1.513 to 9.691 | 100.00 | 100.00 |

## Test for heterogeneity

| Q | 17.3421 |
| --- | --- |
| DF | 6 |
| Significance level | P = 0.0081 |
| I^2^ (inconsistency) | 65.40% |
| 95% CI for I^2^ | 22.34 to 84.59 |

## Publication bias

| Egger's test | |
| --- | --- |
| Intercept | 3.5908 |
| 95% CI | -4.4594 to 11.6411 |
| Significance level | P = 0.3034 |
| Begg's test | |
| Kendall's Tau | 0.2928 |
| Significance level | P = 0.3558 |

Figure 7.2 Forest plot of pooled revision rates of aTSA PAG

# Meta-analysis: proportion

| Variable for studies | studies |
| --- | --- |
| Variable for total number of cases | patients |
| Variable for number of positive cases | Revisions |

| Study | Sample size | Proportion (%) | 95% CI | Weight (%) | |
| --- | --- | --- | --- | --- | --- |
|  |  |  |  | Fixed | Random |
| Favorito et al, 2016 | 22 | 9.091 | 1.121 to 29.161 | 7.32 | 8.67 |
| Grey et al, 2020 | 58 | 3.448 | 0.420 to 11.908 | 18.79 | 17.86 |
| Gutman et al, 2023 | 50 | 2.000 | 0.0506 to 10.647 | 16.24 | 16.15 |
| Ho et al, 2018 | 71 | 0.000 | 0.000 to 5.063 | 22.93 | 20.35 |
| Iannotti et al, 2021 | 50 | 4.000 | 0.488 to 13.714 | 16.24 | 16.15 |
| Kohan et al, 2022 | 35 | 0.000 | 0.000 to 10.003 | 11.46 | 12.47 |
| Stephens et al, 2017 | 21 | 0.000 | 0.000 to 16.110 | 7.01 | 8.35 |
| Total (fixed effects) | 307 | 2.464 | 1.051 to 4.848 | 100.00 | 100.00 |
| Total (random effects) | 307 | 2.557 | 0.901 to 5.027 | 100.00 | 100.00 |

## Test for heterogeneity

| Q | 8.1215 |
| --- | --- |
| DF | 6 |
| Significance level | P = 0.2293 |
| I^2^ (inconsistency) | 26.12% |
| 95% CI for I^2^ | 0.00 to 67.83 |

## Publication bias

| Egger's test | |
| --- | --- |
| Intercept | 1.8280 |
| 95% CI | -3.9955 to 7.6516 |
| Significance level | P = 0.4564 |
| Begg's test | |
| Kendall's Tau | 0.1952 |
| Significance level | P = 0.5382 |
