# Supplementary material for: Outcomes of anatomic versus reverse shoulder arthroplasty for B2 & B3 glenoids with an intact rotator cuff: An updated systematic review and proportional meta-analysis
Source: Shoulder Elbow. 2025 Jul 17;18(3):425–36. doi: 10.1177/17585732251359590 (PMC12274211; doi:10.1177/17585732251359590)
Supplement: sj-docx-7-sel-10.1177_17585732251359590 - Supplemental material for Outcomes of anatomic versus reverse shoulder arthroplasty for B2 & B3 glenoids with an intact rotator cuff: An updated systematic review and proportional meta-analysis [file sj-docx-7-sel-10.1177_17585732251359590.docx]

| Authors | Patients (shoulders), n | Subscapularis takedown | Implant | Method of correction | Additional procedures or notes |
| --- | --- | --- | --- | --- | --- |
| Alentorn-Geli et al, 2018 | 15 (15) | NR | Pegged: Cemented all-poly | NR | - Posterior capsular plication in all |
| Bevan et al, 2023 | 18 (18) | Peel | Pegged: Cemented all-poly | ER |  |
| Chamberlain et al, 2020 | 20 (20) | LTO | Pegged: Cemented all-poly | ER |  |
| Chen et al, 2020 | 22 (22) | LTO/Peel | Metal-backed glenoid | ER |  |
| Chin et al, 2015 | 48 (48) | NR | Pegged or Keeled: Cemented all-poly | ER |  |
| Conyer et al, 2023 | 30 (30) | NR | NR | NR |  |
| Cuff et al, 2023 | 101 (101) | LTO | Pegged: Cemented all-poly | ER |  |
| Egger et al, 2019 | 15 (15) | Tenotomy | Inlay: Cemented all-poly | NR |  |
| Favorito et al, 2016 | 19 (22) | LTO | Pegged: Cemented all-poly | Stepped Augment |  |
| Gallusser et al, 2014 | 17 (19) | NR | Keeled: Cemented all-poly | ER | - 14 ER - 5 BG |
| Grantham et al, 2020 | 43 (45) | LTO | Pegged: Cemented all-poly | NCR |  |
| Grey et al, 2020 | 58 (58) | NR | Pegged: Cemented all-poly | Wedged Augment |  |
| Gutman et al, 2023 | 50 (50) | LTO | Pegged: Cemented all-poly | Stepped Augment |  |
| Habermeyer et al, 2007 | 24 (24) | Tenotomy | Metal-backed glenoid | ER | - 8 BG  - 2 Cemented all-poly |
| Harold et al, 2023 | 33 (34) | LTO | Pegged: Cemented all-poly | ER |  |
| Hinse et al, 2023 | 30 (32) | LTO | Pegged or Keeled: Cemented all-poly | ER |  |
| Ho et al, 2018 | 71 (71) | LTO | Pegged: Cemented all-poly | Stepped Augment |  |
| Hussey et al, 2015 | 78 (78) | LTO | Pegged: Cemented all-poly | ER |  |
| Iannotti et al, 2021 | 50 (50) | LTO | Pegged: Cemented all-poly | Stepped Augment |  |
| Klika et al, 2014 | 11 (11) | NR | NR | NR |  |
| Kohan et al, 2022 | 35 (35) | LTO | Pegged: Cemented all-poly | ER/Stepped Augment |  |
| Leschinger et al, 2017 | 27 (27) | Tenotomy | Keeled: Cemented all-poly | ER |  |
| Magosch et al, 2017 | 68 (68) | NR | NR | NR |  |
| Matsen et al, 2020 | 135 (135) | Peel | Pegged: Cemented all-poly | NCR | - 3 Rotator interval plication |
| Orvets et al, 2018 | 59 (59) | LTO | Pegged: Cemented all-poly | ER |  |
| Pastor et al, 2015 | 4 (4) | Tenotomy | Pegged: Cemented all-poly | NR |  |
| Polisetty et al, 2023 | 101 (101) | Peel | Pegged: Cemented all-poly | ER |  |
| Sheth et al, 2020 | 111 (111) | NR | Pegged: Cemented all-poly | ER |  |
| Stephens et al, 2017 | 21 (21) | NR | Pegged: Cemented all-poly | Stepped Augment |  |
| Walch et al, 2012 | 75 (92) | NR | Pegged: Cemented all-poly | ER | - 10 % Posterior capsular plication  - 7 BG |

**Appendix Table I:** Surgical intervention utilized for aTSA
