# Supplementary material for: Outcomes of anatomic versus reverse shoulder arthroplasty for B2 & B3 glenoids with an intact rotator cuff: An updated systematic review and proportional meta-analysis
Source: Shoulder Elbow. 2025 Jul 17;18(3):425–36. doi: 10.1177/17585732251359590 (PMC12274211; doi:10.1177/17585732251359590)
Supplement: sj-docx-8-sel-10.1177_17585732251359590 - Supplemental material for Outcomes of anatomic versus reverse shoulder arthroplasty for B2 & B3 glenoids with an intact rotator cuff: An updated systematic review and proportional meta-analysis [file sj-docx-8-sel-10.1177_17585732251359590.docx]

**Appendix Table II:** Surgical intervention utilized for rTSA

| First author & year | Patients (shoulders) (n) | Subscapularis takedown | Method of correction | Additional notes |
| --- | --- | --- | --- | --- |
| Alentorn-Geli et al, 2018 | 16 (16) | NR | ER ± BG | - 4 BG |
| Bevan et al, 2023 | 17 (19) | Peel | ± Augment |  |
| Collin et al, 2019 | 27 (27) | Tenotomy | ER ± BG | - 16 BG |
| Cuff et al, 2023 | 93 (93) | LTO | ER ± BG | - 5 BG |
| Gallusser et al, 2014 | 8 (8) | NR | ER ± BG | - 2 BG |
| Harmsen et al, 2017 | 26 (29) | Peel | ER ± BG | - all BG |
| Magosch et al, 2017 | 7 (7) | NR | NR |  |
| Mizuno et al, 2013 | 27 (27) | Tenotomy | ER ± BG | - 10 BG |
| Pettit et al, 2022 | 106 (106) | Peel | ER ± BG | - 5 BG |
| Pharr et al, 2021 | 32 (32) | Tenotomy | NR |  |
| Polisetty et al, 2023 | 101 (101) | Peel | NR |  |
| Waterman et al, 2020 | 20 (20) | NR | NR | - 9 BG |
