# Supplementary material for: Outcomes of anatomic versus reverse shoulder arthroplasty for B2 & B3 glenoids with an intact rotator cuff: An updated systematic review and proportional meta-analysis
Source: Shoulder Elbow. 2025 Jul 17;18(3):425–36. doi: 10.1177/17585732251359590 (PMC12274211; doi:10.1177/17585732251359590)
Supplement: sj-docx-9-sel-10.1177_17585732251359590 - Supplemental material for Outcomes of anatomic versus reverse shoulder arthroplasty for B2 & B3 glenoids with an intact rotator cuff: An updated systematic review and proportional meta-analysis [file sj-docx-9-sel-10.1177_17585732251359590.docx]

**Appendix Table III:** ROM data for included aTSA studies.

| First year & author *(subgroup)* | Patients (shoulders), n | Mean flexion at final FU, (SD) | Δ flexion at final FU, (SD) | Mean external rotation at final FU, (SD) | Δ external rotation at final FU, (SD) | Mean abduction at final FU, (SD) | Δ abduction at final FU, (SD) |
| --- | --- | --- | --- | --- | --- | --- | --- |
| Alentorn-Geli et al, 2018 * | 15 (15) | 164.9 (±13.8) | 77.6 (±26.2) | 61.7 (±19.2) | 52.1 (±31.6) | NR | NR |
| Bevan et al, 2023 * | 18 (18) | 156 (±25) | 35 (±48.4) | 70 (±13) | 24 (±28.5) | 130 (±36) | 61 (±38.37) |
| Chamberlain et al, 2020 | 20 (20) | NR | NR | NR | NR | NR | NR |
| Chen et al, 2020 | 22 (22) | 135 (±20) | 36 (±29.7) | 62 (±20) | 45 (±25) | NR | NR |
| Chin et al, 2015 | 48 (48) | 150 (NR) | 42 (NR) | 45 (NR) | 23 (NR) | 115 (NR) | 30 (NR) |
| Conyer et al, 2023 | 30 (30) | 132.3 (±39.2) | 33.6 (±50.2) | 43.3 (±25.1) | 32.2 (±28.9) | NR | NR |
| Cuff et al, 2023 * | 101 (101) | 152 (NR) | 30 (NR) | 45 (NR) | 19 (NR) | NR | NR |
| Egger et al, 2019 | 15 (15) | NR | NR | 56.1 (±17.9) | NR | NR | NR |
| Favorito et al, 2016 | 19 (22) | 136° (± 26°) | 26° (±49.6 °) | 39.7° (±15.8°) | 29.9° (±30°) | NR | NR |
| Gallusser et al, 2014 * | 17 (19) | 129◦ (NR) | NR | 35◦ (NR) | NR | 125◦ (NR) | NR |
| Grantham et al, 2020 | 43 (45) | NR | NR | NR | NR | NR | NR |
| Grey et al, 2020 – (*B2)* | 46 (46) | 151 (±16) | 52 (±30.8) | 58 (NR) | 37 (NR) | 143 (±25) | 55 (±36) |
| Grey et al, 2020 – (*B3)* | 12 (12) | 154 (±29) | 55 (±38) | 59 (NR) | 32 (NR) | 143 (±25) | 55 (±36) |
| Gutman et al, 2023 – *(B2)* | 41 (41) | 156 (±11.6) | 44 (±30.1) | 41.9 (±6.57) | 20 (±14.4) | NR | NR |
| Gutman et al, 2023 – (*B3)* | 9 (9) | 150 (±9.01) | 50 (±26.1) | 40.3 (±5.34) | 22 (±14.6) | NR | NR |
| Habermeyer et al, 2007 | 24 (24) | NR | NR | NR | NR | NR | NR |
| Harold et al, 2023 | 33 (34) | NR | NR | NR | NR | NR | NR |
| Hinse et al, 2023 | 30 (32) | 151 (±20) | 47 (±37.2) | 45 (±17) | 24 (±24) | 152 (±23) | 64 (±40.6) |
| Ho et al, 2018 | 71 (71) | 160 (NR) | 50 (NR) | 50 (NR) | 30 (NR) | NR | NR |
| Hussey et al, 2015 | 78 (78) | NR | NR | NR | NR | NR | NR |
| Iannotti et al, 2021 | 50 (50) | NR | NR | NR | NR | NR | NR |
| Klika et al, 2014 | 11 (11) | 147.9 (NR) | NR | 61.6 (NR) | NR | NR | NR |
| Kohan et al, 2022 – *(B3 – ST)* | 19 (19) | 149 (NR) | NR | 39 (NR) | NR | NR | NR |
| Kohan et al, 2022 – *(B3 – AG)* | 16 (16) | 148 (NR) | NR | 36 (NR) | NR | NR | NR |
| Leschinger et al, 2017 | 27 (27) | NR | NR | NR | NR | NR | NR |
| Magosch et al, 2017 * | 68 (68) | 149.7 (±25.8) | 54.9 (±44) | 44.8 (±19.2) | 29.1 (±26.5) | 138.9 (±30.8) | 68.5 (±43.6) |
| Matsen et al, 2020 | 135 (135) | NR | NR | NR | NR | NR | NR |
| Orvets et al, 2018 | 59 (59) | NR | NR | NR | NR | NR | NR |
| Pastor et al, 2015 | 4 (4) | NR | NR | NR | NR | NR | NR |
| Polisetty et al, 2023 – (*B2)** | 70 (70) | 143 (±18) | NR | 54 (±19) | NR | NR | NR |
| Polisetty et al, 2023 – (*B3)** | 31 (31) | 140 (±17) | NR | 55 (±14) | NR | NR | NR |
| Sheth et al, 2020 | 111 (111) | NR | NR | 45.5 (±12.1) | 34.8 (±18.7) | NR | NR |
| Stephens et al, 2017 | 21 (21) | 159.3 (NR) | 49.8 (NR) | 48.1 (NR) | 31.9 (NR) | NR | NR |
| Walch et al, 2012 | 75 (75) | 143.3 (NR) | 44.9 (NR) | 37.3 (NR) | 30.3 (NR) | NR | NR |
|  | 1389 (1416) | 148.6 I (790) | 44 (626) | 47.4 (916) | 29.1 (737) | 138 (239) | 55.4 (222) |

Δ, change; FU, follow-up; SD, standard deviation; NR, not reported; ST, standard augment; AG, augmented glenoid; ROM, range of motion; aTSA, anatomic shoulder arthroplasty
* Study includes both rTSA and aTSA
