# Supplementary material for: Outcomes of anatomic versus reverse shoulder arthroplasty for B2 & B3 glenoids with an intact rotator cuff: An updated systematic review and proportional meta-analysis
Source: Shoulder Elbow. 2025 Jul 17;18(3):425–36. doi: 10.1177/17585732251359590 (PMC12274211; doi:10.1177/17585732251359590)
Supplement: sj-docx-10-sel-10.1177_17585732251359590 - Supplemental material for Outcomes of anatomic versus reverse shoulder arthroplasty for B2 & B3 glenoids with an intact rotator cuff: An updated systematic review and proportional meta-analysis [file sj-docx-10-sel-10.1177_17585732251359590.docx]

| First author & year (*subgroup*) | Patients (shoulders), n | Mean flexion at final FU, (SD) | Δ flexion at final FU, (SD) | Mean external rotation at final FU, (SD) | Δ external rotation at final FU, (SD) | Mean abduction at final FU, (SD) | Δ abduction at final FU, (SD) |
| --- | --- | --- | --- | --- | --- | --- | --- |
| Alentorn-Geli et al, 2018 * | 16 (16) | 160 (±22.5) | 73.2 (±29.4) | 53.7 (±34.4) | 43.7 (±36.8) | NR | NR |
| Bevan et al, 2023 * | 17 (17) | 144 (±14) | 52 (±25.2) | 42 (±15) | 23 (±19.8) | 130 (±36) | 61 (±38.6) |
| Collin et al, 2019 – (*B2)* | 15 (15) | 147 (NR) | NR | 18 (NR) | NR | NR | NR |
| Collin et al, 2019 – (*B3)* | 12 (12) | 137 (NR) | NR | 19 (NR) | NR | NR | NR |
| Cuff et al, 2023 * | 93 (93) | 149 (NR) | 24 (NR) | 41 (NR) | 18 (NR) | NR | NR |
| Gallusser et al, 2014 | 8 (8) | 130◦ (NR) | NR | 18◦ (NR) | NR | 125◦ (NR) | NR |
| Harmsen et al, 2017 | 26 (29) | 138.6 (NR) | 41.3 (NR) | 32.8 (NR) | 16.4 (NR) | 130.9 (NR) | 46.5 (NR) |
| Magosch et al, 2017 * | 7 (7) | 137.2 (±22.2) | 60.1 (±47.3) | 27.2 (±17.2) | 11.2 (±24) | 128.9 (±32.2) | 68.9 (±47.6) |
| Mizuno et al, 2013 | 27 (27) | 152 (NR) | 63 (NR) | 27 (NR) | 24 (NR) | NR | NR |
| Pettit et al, 2022 – (*B2)* | 57 (57) | 140.1 (±15.4) | 49.7 (±26) | 56.2 (±17.9) | 31.3 (±20.9) | NR | NR |
| Pettit et al, 2022 – (*B3)* | 49 (49) | 138.4 (±16.1) | 44.1 (±26.5) | 50.1 (±16.5) | 25.6 (±21.5) | NR | NR |
| Pharr et al, 2021 | 32 (32) | 153 (NR) | 69 (NR) | 40 (NR) | 14 (NR) | NR | NR |
| Polisetty et al, 2023 * | 101 (101) | 141 (±16) | 47 (±27.2) | 52 (±18) | 29 (±22.8) | NR | NR |
| Waterman et al, 2020 | 20 (20) | NR | NR | NR | NR | NR | NR |
|  | 480 (485) | 144.1 (460) | 45.7 (425) | 43.4 (460) | 24.3 (425) | 129.5 (58) | 54.5 (50) |

**Appendix Table IV:** ROM data for included rTSA studies.

Δ, change; FU, follow-up; SD, standard deviation; NR, not reported; ROM, range of motion; rTSA, reverse shoulder arthroplasty
* Study includes both rTSA and aTSA
