# Supplementary material for: Outcomes of anatomic versus reverse shoulder arthroplasty for B2 & B3 glenoids with an intact rotator cuff: An updated systematic review and proportional meta-analysis
Source: Shoulder Elbow. 2025 Jul 17;18(3):425–36. doi: 10.1177/17585732251359590 (PMC12274211; doi:10.1177/17585732251359590)
Supplement: sj-docx-11-sel-10.1177_17585732251359590 - Supplemental material for Outcomes of anatomic versus reverse shoulder arthroplasty for B2 & B3 glenoids with an intact rotator cuff: An updated systematic review and proportional meta-analysis [file sj-docx-11-sel-10.1177_17585732251359590.docx]

**Appendix Table V:** PROMs, complications, and revisions data for included aTSA studies.

| First author & year  (*subgroup*) | Patients (shoulders), n | Mean ASES at final FU, points (SD) | Δ ASES at final FU, points (SD) | Mean CS at final FU, points (SD) | Δ CS at final FU, points (SD) | Mean VAS at final FU, points (SD) | Δ VAS at final FU, points (SD) | Other PROMs | Complications rate, n (%) | Revisions rate, n (%) | Notes on revisions |
| --- | --- | --- | --- | --- | --- | --- | --- | --- | --- | --- | --- |
| Alentorn-Geli et al, 2018* | 15 | 91.2 (±6.7) | NR | NR | NR | NR | NR |  | 4 (26) | 0 (0) |  |
| Bevan et al, 2023 * | 18 | 87 (±15) | NR | NR | NR | NR | NR |  | 0 (0) | 0 (0) |  |
| Chamberlain et al, 2020 | 20 | 86.2 (±16.5) | 53.5 (±21.2) | NR | NR | 1.0 (±1.7) | -6.5 (±2.2) |  | 0 (0) | 0 (0) |  |
| Chen et al, 2020 | 22 | 89 (±16.8) | NR | NR | NR | NR | NR |  | 0 (0) | 0 (0) |  |
| Chin et al, 2015 | 48 | NR | NR | NR | NR | NR | NR | Δ QuickDASH: 45 | 1 (2) | 1 (2) | - 1 subscapularis tear → repaired |
| Conyer et al, 2023 | 30 | 84.5 (±14.2) | NR | NR | NR | 1.6 (±2.1) | -7.2 (±2.3) | SSV Postop: 71.3 (±25.3) Δ SSV: 51.3 (±27.51) | 6 (20) | 3 (10) | - 1 glenoid loosening →rTSA  - 1 posterior dislocation → rTSA - 1 cuff failure → rTSA |
| Cuff et al, 2023 * | 101 | 77 (NR) | 36 (NR) | NR | NR | NR | NR | SST postop: 8 (5-11)  Δ SST: 3 | 0 (0) | 10 (8) | - 4 glenoid loosening → rTSA  - 4 cuff failure → rTSA - 1 recurrent posterior subluxation → rTSA  - 1 infection → I/D + rTSA |
| Egger et al, 2019 | 15 | NR | NR | NR | NR | 1.4 (±1.5) | NR | PSS postop: 88.6 (± 9.9)  Δ PSS: 45.4 (±19.76) | NR | NR |  |
| Favorito et al, 2016 | 19 | NR | NR | NR | NR | 1.7 (±2) | -5.6 (±2.7) | WOOS postop: 85.7% (±16.1%)  Δ WOOS: 43.3 (±20.82) | 2 (9) | 2 (9) | - 1 anterior dislocation → LTO repair + larger humeral head replacement - 1 posterior dislocation |
| Gallusser et al, 2014 * | 17 | NR | NR | 65 (NR) | NR | NR | NR | CS postop: 65 (53–77) SST postop: 88 (58–100) SSV postop: 79% (50–95) | 4 (21) | 2 (10.5) | - 1 posterior dislocation → rTSA - 1 infection |
| Grantham et al, 2020 | 43 | 79.6 (±18.3) | 27.1 (±26.5) | NR | NR | NR | NR | SANE postop: 74.7 (±25.6)  Δ SANE: 22.3 (±32.8) | 6 (13.3) | 6 (13.3) | - 2 glenoid loosening → aTSA - 2 subscapularic tear → repaired. - 1 infection - 1 adhesive capsulitis |
| Grey et al, 2020 – (*B2)* | 46 | 89.7 (±15) | 46.9 (±20.5) | 78.4 (NR) | 35.9 (NR) | NR | NR | SST postop: 11.4 (±1.5)  Δ SST: 5.6 (±3.26) | 2 (3.4) | 2 (3.4) | - 2 glenoid loosening |
| Grey et al, 2020 – (*B3)* | 12 | 91.9 (±7) | 53.1 (±13) | 81.3 (NR) | 36.6 (NR) | NR | NR | SST postop: 11.3 (±1.2)  Δ SST: 5.7 (±2.95) |  |  |  |
| Gutman et al, 2023 – *(B2)* | 41 | NR | NR | NR | NR | 0.52 (±0.91) | NR | SANE postop: 95.5 (±5.17) | 1 (2) | 1 (2) | - 1 glenoid loosening |
| Gutman et al, 2023 – (*B3)* | 9 | NR | NR | NR | NR | 0.78 (±0.97) | NR | SANE postop: 90.6 (±5.83) |  |  |  |
| Habermeyer et al, 2007 | 24 | NR | NR | 89.1 (NR) | 39.9 (NR) | NR | NR |  | 1 (4.1) | 2 (8.3) | - 1 glenoid loosening due to bone graft resorption - 1 metal backed glenoid component failure → revised to cemented component |
| Harold et al, 2023 | 33 | 86.0 (±15.5) | 48.2 (±21.9) | NR | NR | 1.0 (±1.5) | -6.1 (±2.3) | Δ SST: 4.7 (±2.6) | 4 (11.7) | 3 (9) | - 1 glenoid loosening → rTSA + BG  - 1 posterior dislocation → revised with eccentric head - 1 large cuff tear → revised to rTSA |
| Hinse et al, 2023 | 30 | NR | NR | 78 (±7) | 36 (±18.4) | NR | NR | SSV postop %: 90 (±11) Δ SSV, %: 50 (±19.42) | 10 (31.2) | 4 (13.3) | - 1 LTO nonunion  - 1 posterior instability → rTSA - 1 glenoid loosening → rTSA - 1 infection → hemiarthroplasty |
| Ho et al, 2018 | 71 | NR | NR | NR | NR | NR | NR | PSS postop: 94 (88-98)  Δ PSS: 64 | 0 (0) | 0 (0) |  |
| Hussey et al, 2015 | 78 | 77.6 (±21.1) | 35 (±27.4) | NR | NR | NR | NR |  | 9 (11.5) | 3 (3.8) | - 2 glenoid loosening - 1 cuff failure |
| Iannotti et al, 2021 - *(B2)* | 29 | NR | NR | NR | NR | NR | NR | PSS postop: 97.4  Δ PSS: 66.8 | 3 (6) | 2 (4) | - 2 LTO failure → revision repair |
| Iannotti et al, 2021 - *(B3)* | 21 | NR | NR | NR | NR | NR | NR | PSS postop: 96.7  Δ PSS: 59.6 |  |  |  |
| Klika et al, 2014 | 11 | NR | NR | NR | NR | NR | NR | Neer result: 7 excellent, 2 satisfactory, & 1 unsatisfactory. | 5 (45) | 2 (16.6) | - 2 glenoid loosening → rTSA |
| Kohan et al, 2022 - *(B3 ST)* | 19 | 85.7 (NR) | NR | NR | NR | NR | NR |  | 6 (17.1) | 0 (0) |  |
| Kohan et al, 2022 - *(B3 AG)* | 16 | 93.3 (NR) | NR | NR | NR | NR | NR |  |  |  |  |
| Leschinger et al, 2017 | 27 | NR | NR | NR | NR | NR | NR | Δ CS: 46.6 (-13 to 98) | NR | 0 (0) |  |
| Magosch et al, 2017 * | 68 | NR | NR | 74 (±16.3) | 45.6 (±21.3) | NR | NR |  | NR | NR |  |
| Matsen et al, 2020 – (B2) | 83 | NR | NR | NR | NR | NR | NR | SST postop: 9.8 (±2.1)  Δ STT: 6.8 (±2.97) SANE postop: 85 (±15)  Δ 45 (±24.21) |  |  |  |
| Matsen et al, 2020 – (B3) | 52 | NR | NR | NR | NR | NR | NR | SST postop: 9.8 (±2.1)  Δ STT: 6.8 (±3.19) SANE postop: 86 (±13)  Δ SANE: 48 (±26.42) | 4 (2.9) | 4 (2.9) | - 4 stiffness → only manipulation under anesthesia |
| Orvets et al, 2018 | 59 | 84.3 (±14) | 48.9 (±19.9) | NR | NR | 1.4 (±1.9) | -6 (±2.5) | Δ SST: 4.6 (±2.4) | 0 (0) | 1 (1.6) | - 1 large cuff tear → rTSA |
| Pastor et al, 2015 | 4 | NR | NR | 58.4 (NR) | NR | NR | NR |  | NR | NR |  |
| Polisetty et al, 2023 * | 101 | 83.9 (±21.4) | 50.3 (±27.4) | NR | NR | 1.2 (±2.5) | -5 (±3.5) |  | 6 (5.9) | 1 (1) | - 1 infection |
| Sheth et al, 2020 | 111 | 88.8 (±18.6) | 49 (±26.2) | 78.4 (±14.9) | 51.6 (±21.1) | NR | NR |  | 8 (7.2) | 6 (5.4) | - 3 posterior instability, 2 revised to rTSA, & 1 with posterior capsular plication and anteverted humeral component - 2 infection → rTSA - 1 glenoid loosening → rTSA |
| Stephens et al, 2017 | 21 | 91 (NR) | 52 (NR) | NR | NR | 0.3 (NR) | -6 (NR) |  |  |  |  |
| Walch et al, 2012 | 75 | NR | NR | 68.8 (NR) | 36.4 (NR) | NR | NR |  | 19 (20.7) | 15 (16.3) | - 6 glenoid loosening; 2 only glenoid removal; 3 bone graft & hemiarthroplasty; one requiring rTSA + BG - 5 posterior instability; 2 rTSA; 3 reorientation of glenoid & plication, all 3 failed and required glenoid removal - 1 subscapularis tear → repaired - 1 capsulitis → arthrolysis - 1 chronic shoulder pain → biceps tenotomy - 1 impingement syndrome → subacromial decompression |
|  |  | 84.3 (745) | 43.9 (625) | 75.6 (387) | 42.8 (366) | 1.13 (348) | -5.79 (283) |  | 101 (7.6%) | 70 (5.2%) |  |

Δ, change; PROMs, patient-reported outcome measures; ASES, American Shoulder and Elbow Surgeons; FU, follow-up; SD, standard deviation; aTSA, anatomic shoulder arthroplasty; rTSA, reverse shoulder arthroplasty; NR, not reported; SST, simple shoulder test; PSS, Penn Shoulder Score; VAS, Visual Analog Scale; SPADI, Shoulder Pain and Disability Index; SSV, subjective shoulder value; SANE, Single Assessment Numeric Evaluation; WOOS, Western Ontario Shoulder Score; QuickDASH, Quick Disabilities of Arm, Shoulder & Hand.
