# Supplementary material for: Outcomes of anatomic versus reverse shoulder arthroplasty for B2 & B3 glenoids with an intact rotator cuff: An updated systematic review and proportional meta-analysis
Source: Shoulder Elbow. 2025 Jul 17;18(3):425–36. doi: 10.1177/17585732251359590 (PMC12274211; doi:10.1177/17585732251359590)
Supplement: sj-docx-12-sel-10.1177_17585732251359590 - Supplemental material for Outcomes of anatomic versus reverse shoulder arthroplasty for B2 & B3 glenoids with an intact rotator cuff: An updated systematic review and proportional meta-analysis [file sj-docx-12-sel-10.1177_17585732251359590.docx]

| First author & year (*subgroup*) | Patients (shoulders), n | Mean ASES at final FU, points (SD) | Δ ASES at final FU, points (SD) | Mean CS at final FU, points (SD) | Δ CS at final FU, points (SD) | Mean VAS at final FU, points (SD) | Δ VAS at final FU, points (SD) | Other PROMs | Complications rate, n (%) | Revisions rate, n (%) | Notes on revisions |
| --- | --- | --- | --- | --- | --- | --- | --- | --- | --- | --- | --- |
| Alentorn-Geli et al, 2018 * | 16 (16) | 80.3 (±14.3) | NR | NR | NR | NR | NR |  | 0 (0%) | 0 (0%) |  |
| Bevan et al, 2023 * | 17 (17) | 93 (±9) | NR | NR | NR | NR | NR |  | 0 (0%) | 0 (0%) |  |
| Collin et al, 2019 – (*B2)* | 15 (15) | NR | NR | 73 (NR) | NR | NR | NR | SSV postop: 79% (NR) | NR | NR |  |
| Collin et al, 2019 – (*B3)* | 12 (12) | NR | NR | 66 (NR) | NR | NR | NR | SSV postop: 78% (NR) |  |  |  |
| Cuff et al, 2023 * | 93 (93) | 80 (NR) | 42 (NR) | NR | NR | NR | NR | Δ SST: 5 (NR) | 3 (3.2%) | 2 (2%) | - 1 dislocation → revised to larger glenosphere and constrained liner -1 infection |
| Gallusser et al, 2014 | 8 (8) | NR | NR | 65 (NR) | NR | NR | NR |  | 0 (0%) | 0 (0%) |  |
| Harmsen et al, 2017 | 26 (29) | 87.9 (NR) | 54 (NR) | NR | NR | 0.3 (NR) | -6.4 (NR) | Δ SANE: 59.5 (NR) | 2 (7.6%) | 1 (3.4%) | - 1 infection |
| Magosch et al, 2017 * | 7 (7) | NR | NR | 72.7 (±21.1) | 52.6 (±25.4) | NR | NR |  | NR | NR |  |
| Mizuno et al, 2013 | 27 (27) | NR | NR | 76 (NR) | 45 (NR) | NR | NR |  | 4 (14.8%) | 1 (3.7%) | - 1 baseplate failure revised to hemiarthroplasty |
| Pettit et al, 2022 – (*B2)* | 57 (57) | 88.8 (±13.2) | 52.7 (±21.5) | NR | NR | 0.49 (±1.1) | -5.51 (±2.73) | Δ SANE: 61.1 (±23.7) | 3 (2.8%) | 1 (0.9%) | 1 baseplate failure → revision |
| Pettit et al, 2022 – (*B3)* | 49 (49) | 91 (±9.2) | 56 (±17.8) | NR | NR | 0.44 (±1.1) | -5.46 (±2.28) | Δ SANE: 60.6 (±21.5) |  |  |  |
| Pharr et al, 2021 | 32 (32) | 78.7 (NR) | 36.9 (NR) | NR | NR | 0.9 (NR) | -4.8 (NR) |  | 2 (6.2%) | 1 (3.%) | - 1 infection |
| Polisetty et al, 2023 * | 101 (101) | 89.8 (±11.7) | 55 (±19.5) | NR | NR | 0.5 (±1) | -5.5 (±2.51) |  | 4 (3.9%) | 1 (0.9%) | - 1 baseplate revision due to trauma |
| Waterman et al, 2020 - *(B2)* | 13 (13) | NR | 49.17 (±25.3) | NR | NR | NR | -4.38 (±3.03) |  | NR | NR |  |
| Waterman et al, 2020 - *(B3)* | 7 (7) | NR | 50.92 (±23.2) | NR | NR | NR | -3.97 (±0.973) |  |  |  |  |
|  | 480 (485) | 86.1 (391) | 49.7 (358) | 72 (69) | 28.7 (34) | 0.52 (265) | -5.41 (265) |  | 19 (4.4) | 7 (1.8) |  |

**Appendix Table VI:** PROMs, complication, and revision data for included rTSA studies.

Δ, change; PROMs, patient-reported outcome measures; ASES, American Shoulder and Elbow Surgeons; CS, constant score; FU, follow-up; SD, standard deviation; rTSA, reverse shoulder arthroplasty; NR, not reported; SST, simple shoulder test; VAS, Visual Analog Scale; SSV, subjective shoulder value; SANE, Single Assessment Numeric Evaluation.
