# Supplementary material for: Outcomes of anatomic versus reverse shoulder arthroplasty for B2 & B3 glenoids with an intact rotator cuff: An updated systematic review and proportional meta-analysis
Source: Shoulder Elbow. 2025 Jul 17;18(3):425–36. doi: 10.1177/17585732251359590 (PMC12274211; doi:10.1177/17585732251359590)
Supplement: sj-docx-13-sel-10.1177_17585732251359590 - Supplemental material for Outcomes of anatomic versus reverse shoulder arthroplasty for B2 & B3 glenoids with an intact rotator cuff: An updated systematic review and proportional meta-analysis [file sj-docx-13-sel-10.1177_17585732251359590.docx]

| **Author** | **MINORS score (/16 or /24)** | |
| --- | --- | --- |
| Alentorn-Geli 2018 | **14** | /24 |
| Bevan 2023 | **13** | /24 |
| Chamberlain 2020 | **10** | /16 |
| Chen 2020 | **17** | /24 |
| Chin 2015 | **15** | /24 |
| Collin. 2019 | **10** | /16 |
| Conyer 2023 | **18** | /24 |
| Cuff 2023 | **17** | /24 |
| Egger 2019 | **18** | /24 |
| Favorito 2016 | **14** | /16 |
| Gallusser 2014 | **12** | /24 |
| Grantham 2020 | **10** | /16 |
| Grey 2020 | **14** | /24 |
| Gutman 2023 | **16** | /24 |
| Habermeyer 2007 | **14** | /24 |
| Harmsen 2017 | **10** | /16 |
| Harold 2023 | **11** | /16 |
| Hinse 2023 | **12** | /16 |
| Ho 2018 | **11** | /16 |
| Hussey 2015 | **12** | /24 |
| Iannotti 2021 | **15** | /24 |
| Klika 2014 | **13** | /16 |
| Kohan 2022 | **12** | /24 |
| Leschinger 2017 | **10** | /16 |
| Magosch 2017 | **10** | /16 |
| Matsen 2020 | **10** | /16 |
| Mizuno 2013 | **9** | /16 |
| Orvets 2018 | **12** | /16 |
| Pastor 2015 | **14** | /24 |
| Pettit 2022 | **11** | /16 |
| Pharr 2021 | **20** | /24 |
| Polisetty 2023 | **17** | /24 |
| Sheth 2020 | **18** | /24 |
| Stephens 2017 | **12** | /16 |
| Walch 2012 | **14** | /16 |
| Waterman 2020 | **13** | /24 |

**Appendix Table VII:** Quality assessment of included studies using MINORS

* MINORS; Methodological Index for Non-Randomized Studies,
